# Dual RNA-seq analysis of in vitro infection multiplicity and RNA depletion methods in *Chlamydia*-infected epithelial cells

**DOI:** 10.1038/s41598-021-89921-x

**Published:** 2021-05-17

**Authors:** Regan J. Hayward, Michael S. Humphrys, Wilhelmina M. Huston, Garry S. A. Myers

**Affiliations:** 1 The iThree Institute, Faculty of Science, University of Technology Sydney, Sydney, Australia; 2 Institute for Genome Sciences, University of Maryland School of Medicine, Baltimore, MD USA; 3 School of Life Sciences, Faculty of Science, University of Technology Sydney, Sydney, Australia; 4 Helmholtz Centre for Infection Research (HZI), Helmholtz Institute for RNA-based Infection Research (HIRI), Würzburg, Germany

**Keywords:** Computational biology and bioinformatics, Gene regulatory networks

## Abstract

Dual RNA-seq experiments examining viral and bacterial pathogens are increasing, but vary considerably in their experimental designs, such as infection rates and RNA depletion methods. Here, we have applied dual RNA-seq to *Chlamydia trachomatis* infected epithelial cells to examine transcriptomic responses from both organisms. We compared two time points post infection (1 and 24 h), three multiplicity of infection (MOI) ratios (0.1, 1 and 10) and two RNA depletion methods (rRNA and polyA). Capture of bacterial-specific RNA were greatest when combining rRNA and polyA depletion, and when using a higher MOI. However, under these conditions, host RNA capture was negatively impacted. Although it is tempting to use high infection rates, the implications on host cell survival, the potential reduced length of infection cycles and real world applicability should be considered. This data highlights the delicate nature of balancing host–pathogen RNA capture and will assist future transcriptomic-based studies to achieve more specific and relevant infection-related biological insights.

## Introduction

Dual species transcriptomic experiments (dual RNA-seq) allow multiple organisms to be simultaneously analysed from within the same sample, such as host and bacterial transcripts during an infection^1^. The number of dual RNA-seq experiments are increasing, with the underlying experimental designs and infection conditions often varying significantly, particularly in infection rates and RNA depletion methods^2–7^.

Here, we have applied dual RNA-seq to human epithelial cells subjected to the bacteria *Chlamydia trachomatis*, which is an obligate intracellular, human-specific bacterial pathogen that causes trachoma and urogenital infections^8–10^. Ocular infections cause trachoma (infectious blindness), typically in disadvantaged communities, and is the leading cause of preventable blindness worldwide^9,10^; while genital infections are the most prevalent sexually transmitted infection (STI) worldwide^9^. If infections are left untreated they can become problematic leading to more complex disease outcomes including ectopic pregnancy and infertility^11,12^. Diagnosed chlamydial infections can successfully be treated with antibiotic therapy, however asymptomatic infections are common^13,14^ and thus challenging to treat. Genome-wide transcriptomic studies have explored gene expression from infected host cells and chlamydial-specific expression, either separately or simultaneously^15–23^. The previous chlamydial-based dual RNA-seq experiment encompassed an experimental design that used a multiplicity of infection (MOI) of 1, while their depletion technique removed rRNAs in all samples, followed by subjecting half of these libraries to polyA depletion to further enrich chlamydial transcripts. Although two depletion methods were used, it is uncertain if this increased the abundance of chlamydial transcripts. Additionally, an MOI of 1 at an early time point highlighted low capture rates of chlamydial transcripts^15^.

In general, host-based RNA-seq experiments in an infection setting will typically attempt to achieve a ratio of 1 infectious entity per host cell. This ratio is referred to as the MOI, with an MOI of 1 indicating a 1:1 ratio, and is frequently used to assess baseline changes in both organisms without any directional bias. RNA-seq and microarray experiments that have focused on chlamydial infection have utilised a range of MOIs ranging from 1^24^ to 100^16,20^; with higher ratios helping to exaggerate and highlight the chlamydial impact. However, too high an MOI and the whole monolayer of cells dies before the infection can proceed. In addition, a higher MOI will likely affect the developmental cycle due to the underlying stress this places on host cells^25,26^.

**Figure 1:**
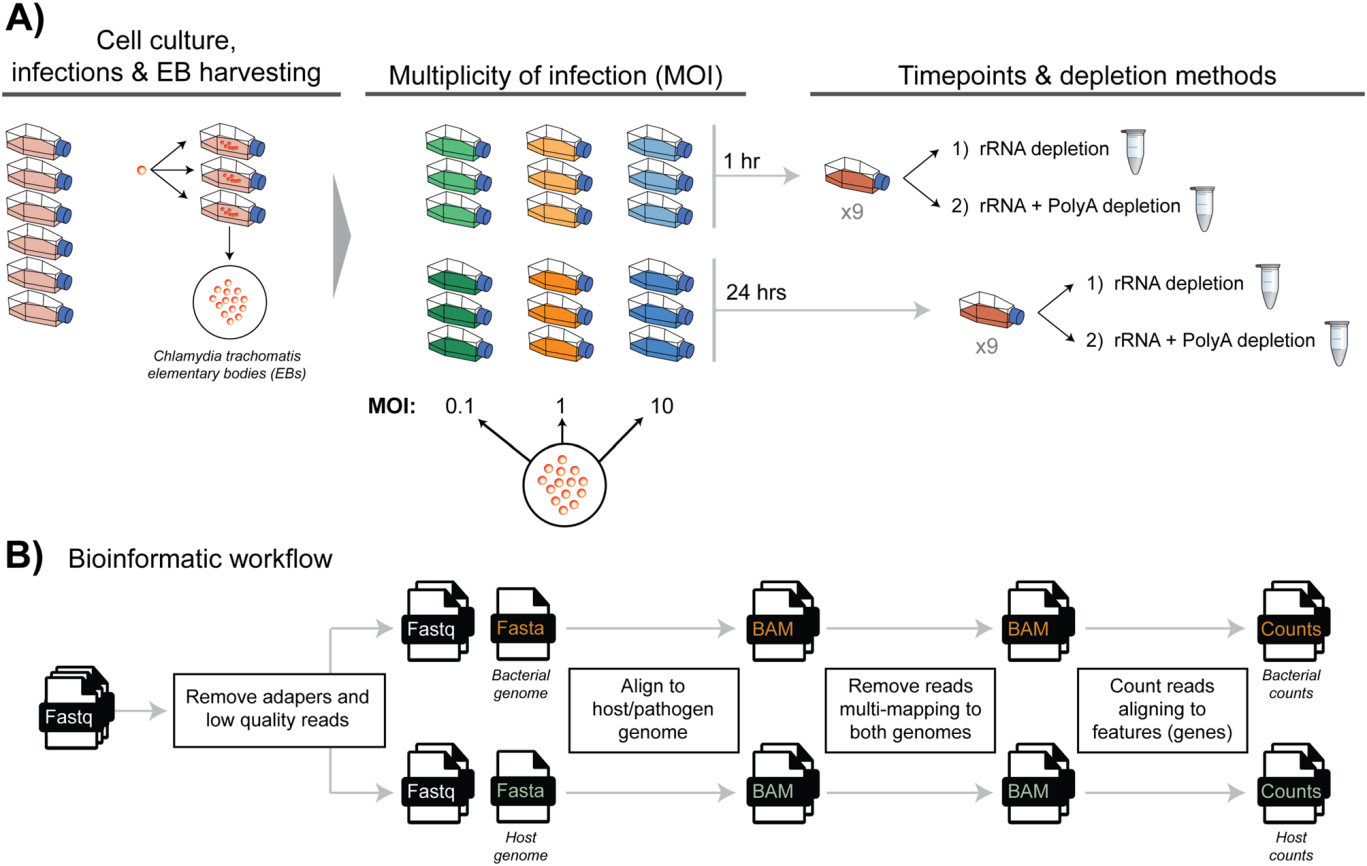
Experimental process, design and bioinformatic workflow. (**A**) The process of growing cell cultures and harvesting (elementary bodies) EBs to use for downstream experiments is a time-consuming process spanning multiple days. The resulting EBs were used for three different infection ratios (multiplicity of infection) of 0.1, 1 and 10. After infections, samples were left for 1 h and 24 h. Each replicate was then prepared with rRNA or rRNA plus PolyA depletion, generating 32 samples in total. (**B**) An overview of the bioinformatic steps to separate unique host and chlamydial reads from initial Fastq sequence files.

In this experiment, both host and chlamydial gene expression were examined applying dual-RNA-seq to in vitro* C. trachomatis*-infected HEp-2 epithelial cells. The first aim was to understand the influence different MOIs have on sequence capture rates, but also the transcriptional variation from *Chlamydia* and the host-cell. The second aim attempted to improve the enrichment of chlamydial reads by comparing different RNA depletion methods. To address these questions, two time points were chosen covering the chlamydial developmental cycle (1 and 24 h), with each time point split into three MOIs (0.1, 1 and 10), each in triplicate. Each of these biological replicates (16 samples) were split in half, where one library was prepared solely with rRNA depletion, while the second was prepared with rRNA depletion followed by polyA depletion (Fig. 1A).

## Results

### Quantifying expression differences between host and chlamydial reads

Dual RNA-seq was applied to *C. trachomatis* serovar E-infected human HEp-2 epithelial cells in triplicate at 1 and 24 h post-infection (hpi). Within each time point, three MOIs were used (0.1, 1 and 10), in addition to two depletion methods (1) rRNA depletion, (2) rRNA depletion and polyA depletion; totalling 36 samples across the experimental design (Fig. 1A).

**Figure 2:**
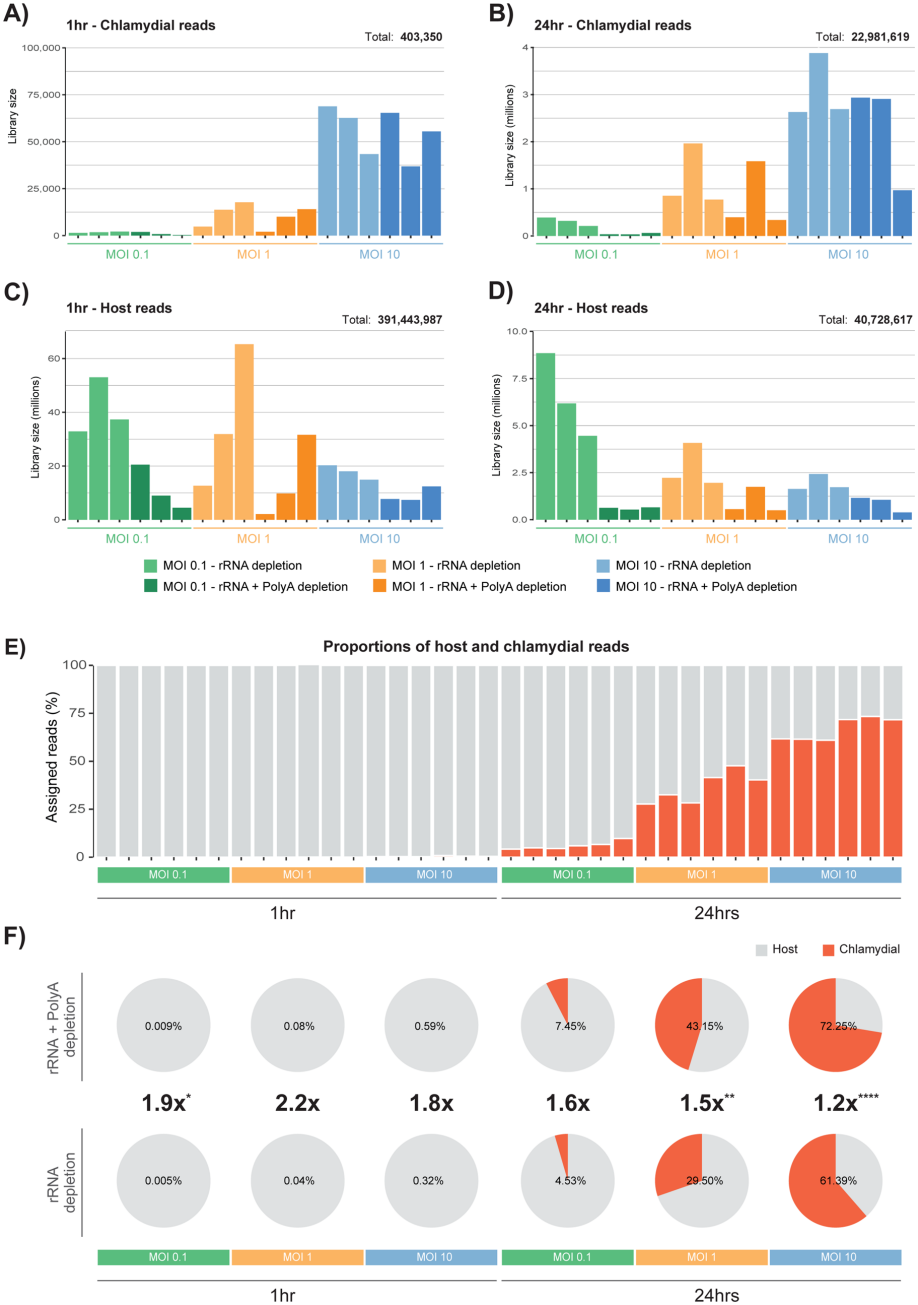
Human and chlamydial mapped reads. The number of mapped sequence reads uniquely assigned to both human and chlamydial genomes*.* Green bars represent an MOI of 0.1, orange 1, and blue 10. Light shaded colours represent the rRNA depletion method, while darker shades represent rRNA and polyA depletion methods combined. (**A**) At 1 h, capturing chlamydial reads is challenging, particularly at MOIs of 0.1 and 1. When using an MOI of 10, a substantial increase in captured chlamydial reads is seen. (**B**) At 24 h the number of captured reads dramatically increases from 1 h, but follows a similar distribution with a spike of reads at an MOI of 10. (**C**) The large increase in assigned host reads at 1 h was to try and capture as many chlamydial transcripts as possible, as they are known to be in very low quantities this early in the developmental cycle. (**D**) As the MOI increases at 24 h, the number of assigned host reads declines. (**E**) By showing the proportion of host (grey) and chlamydial (red) reads across the experimental design, we gradually see an increase of chlamydial reads as the MOI and developmental cycle increases. (**F**) Comparing the proportion of host and chlamydial reads between depletion methods (percentages and corresponding ratios are associated with chlamydial reads), showing that we were able to increase the capture efficiency of chlamydial transcripts at both time points and across MOIs. Statistical significance was calculated using a two-tailed Student’s t-test (*P < 0.05, **P < 0.01, ***P < 0.001 and ****P < 0.0001).

Capture rates of chlamydial reads at early time points is challenging due to limited biological activity, where the majority of transcripts in a sample (> 99%) will be associated with the host^15^. To separate out host and chlamydial-specific reads, we separately aligned to both genomes and removed reads which multi-mapped to both (Fig. 1B). We increased the sequencing depth at 1 h (> sixfold) (Supplementary Table 1) to try and capture more chlamydial reads; assigning 391,847,337 reads at 1 h compared to 63,710,236 reads at 24 h (Supplementary Table 2). Even with this greater depth of sequencing at 1 h, the number of chlamydial reads was still quite low; especially at an MOI of 0.1 with an average of 1,407 reads across the six replicates. However, as the MOI increases, we do see an increase in chlamydial transcripts, with average assigned reads of 10,392 (MOI 1), and 55,426 (MOI 10) (Fig. 2A). At 24 h we see an expected increase in the number of chlamydial reads, following a similar trend with 1 h, where the number of assigned reads increases as the MOI increases (Fig. 2B). The number of assigned host reads tends to vary more than the chlamydial reads, particularly between depletion methods of the same MOI (Fig. 2C,D). This is likely due to the increased variety of host transcripts resulting from post-transcriptional modifications, such as polyadenylation, which does not occur in bacterial systems.

When examining the proportions of host and chlamydial reads together across the experimental design, we see that 1 h is dominated by host reads, while at 24 h we see a gradual increase of chlamydial reads as the MOI increases. Surprisingly, at 24 h with an MOI of 10, the proportion of chlamydial reads across all replicates is over 60% (Fig. 2E).

### Combining depletion methods increases yield of bacterial transcripts

By combining two depletion methods (rRNA depletion and polyA depletion), we had anticipated capturing additional chlamydial reads. The addition of polyA depletion should theoretically remove any polyadenylated host transcripts, thereby increasing the number of chlamydial transcripts to be captured and sequenced.

Overall, we see an increase in chlamydial reads when combining depletion methods, with three of the six conditions showing a significant increase (1 h:MOI:0.1 p-value 1.3 × 10^–3^, 24 h:MOI:1 p-value 7.5 × 10^–3^ and 24 h:MOI:10 p-value 5.2 × 10^–5^). Even at 1 h when there are limited transcripts circulating within the cell, we still see an average increase of 2.0x. At 24 h when more chlamydial transcripts are being expressed, we see an average increase of 1.4× more reads. Interestingly, at 24 h as the MOI increases, the capture efficiency begins to decline slightly from 1.6 × to 1.2x (Fig. 2F).

### Differences in chlamydial expression between depletion methods

**Figure 3:**
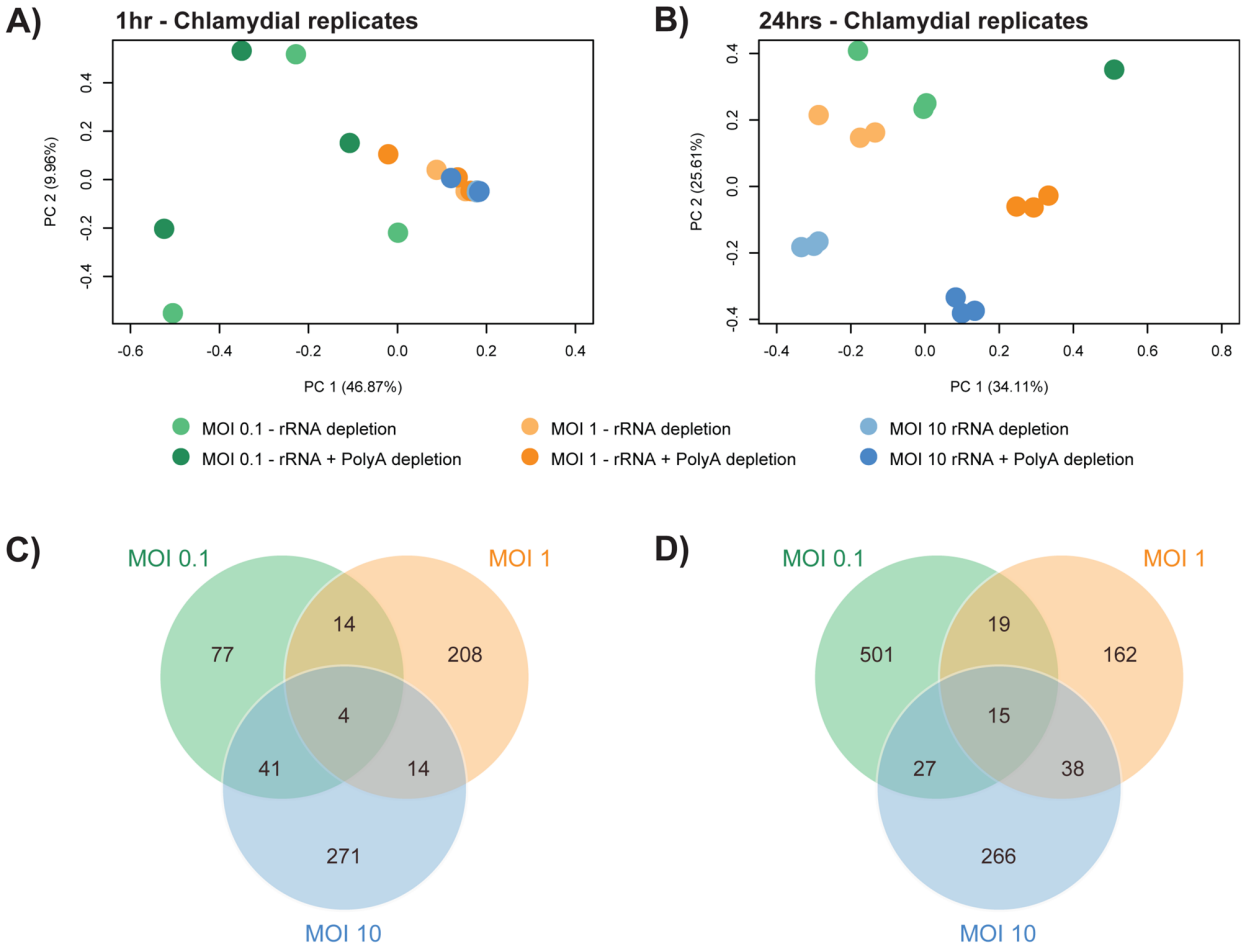
Chlamydial-based expression differences between depletion methods. (**A**) PCA bi-plots of chlamydial-specific reads separated by MOI and depletion method. At 1 h, minimal separation is seen apart from at an MOI of 0.1 where replicates appear to not group or cluster together (due to low assignment of reads). (**B**) At 24 h, we see clustering of depletion-specific replicates, but separation of different depletion methods at each MOI. (**C**) To further explore the separation between depletion methods, we extracted the top 5% of genes driving variation from PC1 and PC2 at each MOI. At 1 h and 24 h (**D**), we see subsets of genes overlapping MOIs in addition to MOI-specific subsets; highlighting that the inclusion of polyA depletion increased the capture rate, but still captured a wide array of chlamydial reads.

PCA bi-plots were created to compare the expression profiles across replicates from both depletion methods. At 1 h, we see minimal separation at an MOI of 1 and 10 compared to 0.1 where replicates appear separated and not grouped by depletion method as expected (Fig. 3A). However, none of the replicates were considered outliers using a robust statistical approach as outlined in the methods. We therefore attribute this variability to the low number of chlamydial reads present at an MOI of 0.1 as identified earlier. At 24 h a distinct separation between depletion methods within each MOI can be easily visualised (Fig. 3B).

To understand if the variability between depletion methods is driven by a small subset of highly expressed genes, or an assortment of genes, we extracted the top 5% of genes driving the underlying variation at PC1 and PC2 for each MOI (Fig. 3C,D). At both time points we see subsets of genes specific to each MOI, indicating that each MOI exhibits a slightly different chlamydial response. In addition, overlapping genes highlight that the variation between depletion methods was also captured and overlaps considerably. Therefore, the inclusion of polyA depletion increases bacterial reads and does not seem to be driven by small subsets of highly expressed transcripts, but allows for a wide array of transcripts to be captured.

### The removal of polyA transcripts increases non-protein coding host gene expression

**Figure 4:**
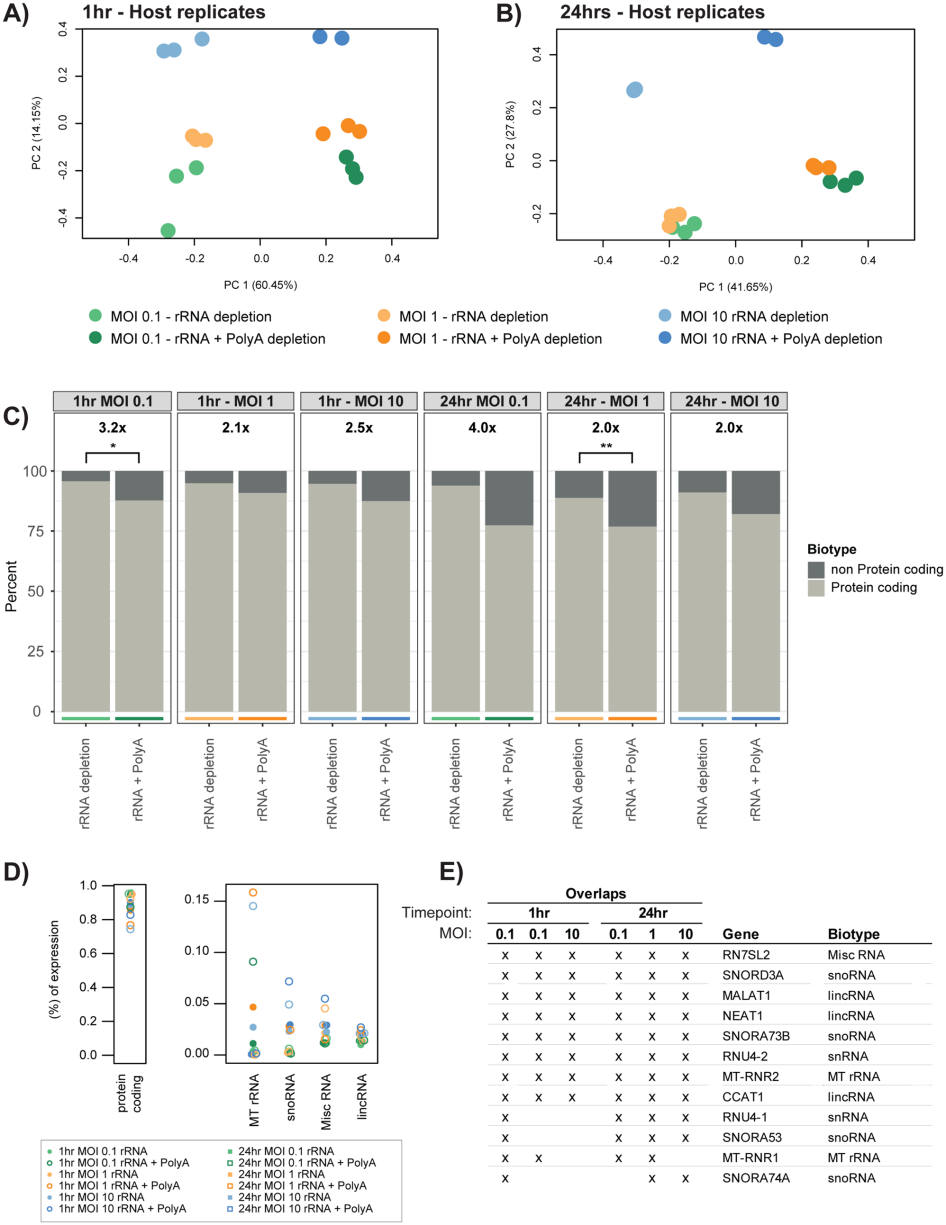
Host-based expression differences between depletion methods. (**A**) PCA bi-plots of host-specific reads. 1 h shows tight grouping between replicates, but separation at each MOI and depletion method. (**B**) Similar clustering to 1 h, but with an MOI of 10 much further separated. (**C**) Comparisons of the proportions of protein coding and non-protein coding gene expression between depletion methods. Values above the plots show the increase in non-protein coding expression when combining rRNA and polyA depletion. Overall, an increase in non-protein coding expression is observed when combining depletion methods. Statistical significance was calculated using a two-tailed Student’s t-test (*P < 0.05, **P < 0.01, ***P < 0.001 and ****P < 0.0001). (**D**) The breakdown of the overall proportions of gene expression for all conditions. The majority of expression is from protein coding genes, while non-protein coding genes are dominated by four biotypes, with MT rRNAs the most highly expressed. (**E**) To observe highly expressed non-protein coding genes that are observed across the different experimental conditions, we extracted the top 200 expressed genes from each MOI, highlighting which genes overlapped 3 or more conditions.

Examining PCA bi-plots for host reads show tight clustering between replicates, but also highlights the separation between depletion methods (Fig. 4A,B). Extracting the underlying genes contributing the variation at PC1 and PC2, numerous non-coding genes were identified. To calculate the percent of protein coding versus non-protein coding expression, gene expression was averaged across replicates after separation by time point, MOI and depletion method (Fig. 4C). Across both time points we see an average of 2.6 × more non-protein coding expression when combining rRNA and polyA depletion, with two significant conditions (1 h:MOI:0.1 p-value 4.4 × 10^–2^ and 24 h:MOI:10 p-value 3.4 × 10^–3^) (Fig. 4C, Supplementary Table 3). We can see that the majority of expression comes from protein-coding genes (Fig. 4C). However, as identified in the PCA plots earlier, non-protein coding expression contributed to the separation of depletion methods. By characterising the most common non-protein coding biotypes (Supplementary Table 4), we see mitochondrial rRNA (MT rRNA), small nucleolar RNAs (snoRNA), miscRNA and long intergenic non-coding (lincRNA); but without any visible trends separating time points, depletion methods or MOI (Fig. 4D).

To identify potentially influential non-protein coding genes, we used the top 200 expressed genes from both depletion methods and extracted a subset of genes that occur frequently (across 3 or more conditions) (Fig. 4E). Of the 12 genes identified, 5 were snoRNAs which are involved with RNA modifications, and are among the most highly abundant non-coding RNAs (ncRNAs) in the nucleus^27^. The MT-RNR1 (12S RNA) and MT-RNR2 (16S RNA) genes encode the two rRNA subunits of mitochondrial ribosomes, and are generally always highly expressed within eukaryotic cells^28^. LincRNAs include CCAT1, which is linked to cell growth and regulation of EGFR^29^, while MALAT1 and NEAT1 co-localise to hundreds of genomic loci, predominantly over active genes^30^.

### Increasing infection highlights minimal changes to highly expressed host and chlamydial genes

To determine whether the host or chlamydial transcriptional-profile changes in relation to the ratio of EBs per cell, highly expressed genes were compared against an MOI of 1. Chlamydial transcripts were examined from the combined depletion replicates, as more transcripts were captured (Fig. 2F), thus giving a more representative profile. Host reads were taken from just the rRNA depleted replicates, as these were shown to contain more of an accurate representation of protein coding and non-protein coding genes (Fig. 4).

**Figure 5:**
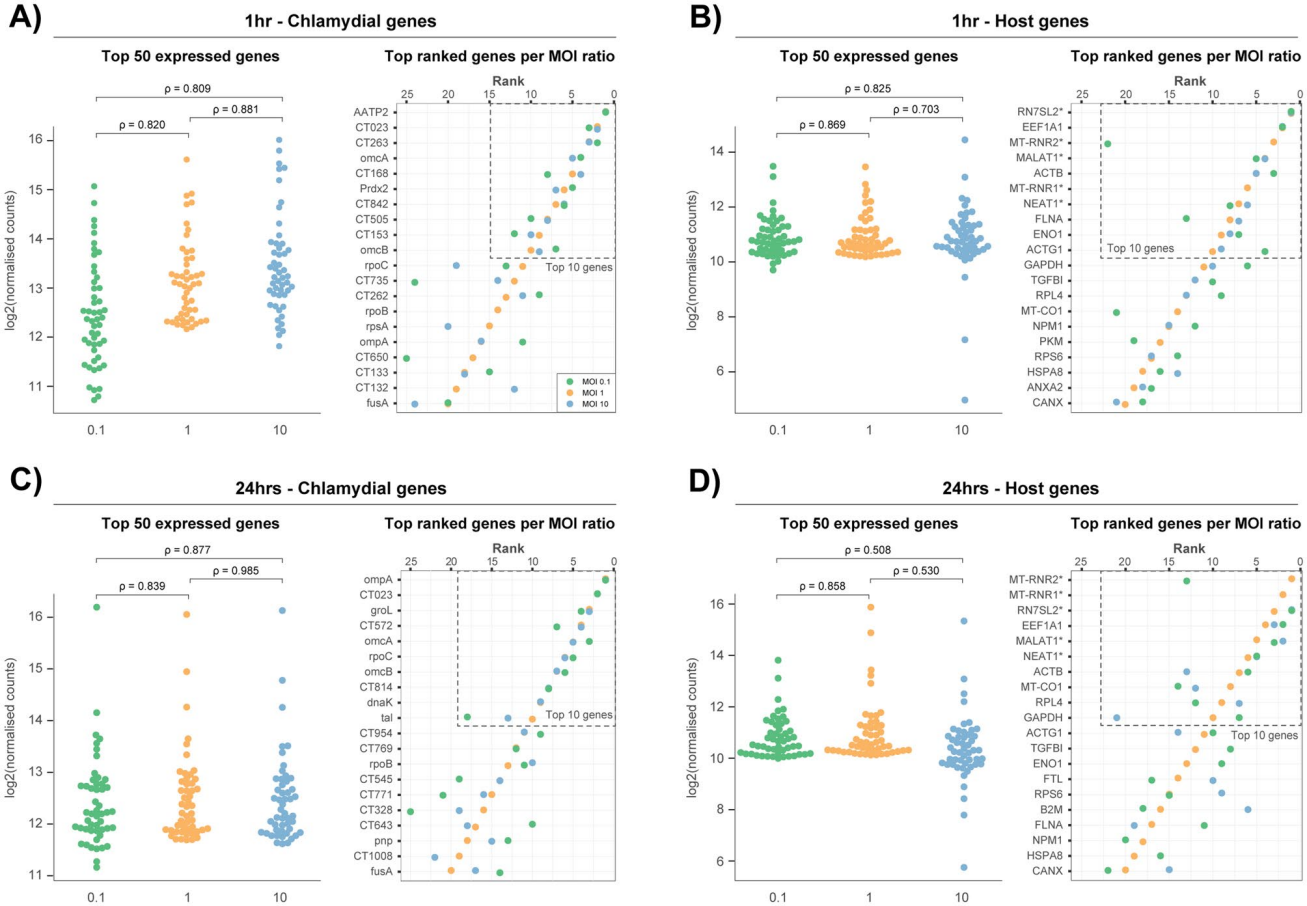
Top 25 expressed host and chlamydial genes across MOIs. Each quadrant contains two graphs: the first showing the top 50 expressed genes across each MOI ( taken from an MOI of 1), with correlations calculated using Spearman’s rho (ρ); while the second graph ranks those same genes for direct comparison. (**A**) Chlamydial expression at 1 h shows a slight upwards trend as the MOI increases, with high similarity in the expression and ranked order. (**B**) Host expression at 1 h shows consistent expression across MOIs apart from two mitochondrial genes (MT-RNR1 and MT-RNR2) with low expression at an MOI of 10. Rankings of the top ten genes are also highly similar. (**C**) At 24 h, chlamydial expression appears to be less influenced by MOI. (**D**) Host expression at 24 h shows high similarity between MOIs 0.1 and 1, whereas an MOI of 10 has a more varied range of expression. Ranked genes highlight the same trend.

Each of the four panels (Fig. 5) contains two graphs. The first graph contains the top 50 expressed genes taken from an MOI of 1 compared against MOIs 0.1 and 10 (Supplementary File 1), using Spearman’s rho (ρ) for correlations. The second graph shows the ranked-positions of these top expressed genes. At 1 h, there is slightly less chlamydial expression at an MOI of 0.1, and slightly more expression when additional EBs are introduced at an MOI of 10. Even with the slight differences in expression, all three MOIs show a high correlation (ρ > 0.8) (Fig. 5A). The ranking chart to the right confirms the high correlation by showing that 9/10 of the top expressed genes remain the same across the three MOIs. The top 25 genes from the host’s response at 1 h share highly similar expression profiles (Fig. 5B); with only two mitochondrial-based genes (MT-RNR1 and MT-RNR2) at an MOI of 10, reducing the similarity with lower expression. The ranking chart shows 7/10 top expressed genes remaining constant across the three MOIs, similar to the chlamydial profile. At 24 h, similar expression profiles of the top 25 expressed chlamydial genes can be seen from the high correlations (ρ > 0.83), irrespective of MOI (Fig. 5C). Rankings are also similar, with only slight variations in the top ten genes, and 16/20 of the top expressed genes remain identical. The host expression profile at 24 h is consistent at an MOI of 1 and 0.1 (ρ = 0.858), whereas the expression pattern is more widely distributed at an MOI of 10 with much lower correlations compared to the lower MOIs; again with MT-RNR1 and MT-RNR2 exhibiting lower expression (Fig. 5D). Although the top ranked genes exhibit more variability within their rankings compared to 1 h, 90% of the top expressed host genes appear at both time points. Functional characterisation of the genes shows their involvement with general cell-based growth events, such as ribosomal-based processes, metabolism, and cytoskeletal components (Supplementary File 2). Many top ranked host genes are also non-protein coding as identified by an asterisk (*). However, with limited annotation available, their characterisation in to infection-association functions are limited. Of the annotated non-protein coding genes, they appear to be involved with general cell regulatory processes. Only seven chlamydial genes overlap both time points, which was anticipated, as two different biological events are occurring at these times, including infection mechanisms at 1 h, and growth-related processes at 24 h. Characterisation of these overlapping genes identifies membrane proteins and transcription/translation machinery, which are needed throughout the developmental cycle (Supplementary File 2).

### Comparative analysis between MOIs show increased expression of inflammatory and immune-based host genes

**Figure 6:**
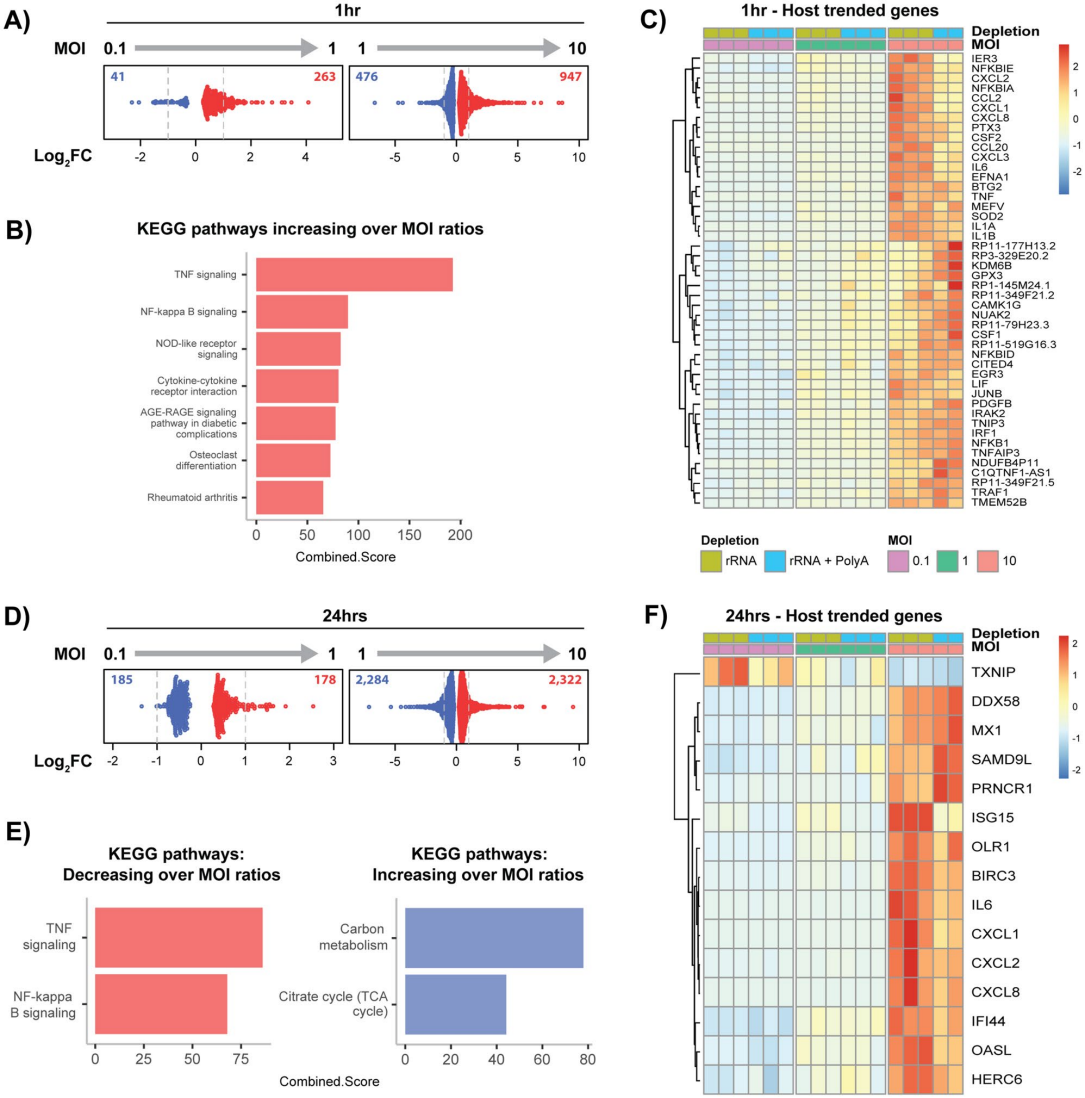
Comparison of differentially expressed host genes across MOIs. Each time point is separated into three panels: the first panel shows the range of fold-changes (each dot representing a gene) after differential expression (FDR < 0.05). KEGG pathways are enriched for genes which exhibited a continued increase or decrease in expression as the MOI increases. Heatmaps show trended genes, which had a fold-change > 2 and followed the same regulation pattern across MOIs. (A) At 1 h, we see more up-regulated genes at both comparisons. (**B**) Enriched pathways highlight that as the MOI increases, we see increased expression of immune and inflammatory responses. (**C**) At 1 h, 46 up-regulated ‘trended-genes’ further highlight the association with the immune system with over 50% of genes grouped in to cytokine signalling, chemokines and interleukins. (**D**) At 24 h the numbers of up and down-regulated genes is much more even (49% and 50%) than 1 h. (**E**) The top two up-regulated pathways are repeated at both time points (*TNF signalling* and *NF-kappa B signalling*), while down-regulated pathways are associated with metabolism and likely indicate cells shifting into defence mode as infection increases. (**F**) Trended-genes further highlight general immune-based genes, in addition to viral and ubiquitin-related immune responses.

To observe and compare how the host expression responded to increased infection, we examined differentially expressed (DE) genes comparing MOIs. At 1 h, the majority of genes (87% from 0.1 to 1, and 67% from 1 to 10) exhibited an increase in regulation as the MOI increased (Fig. 6A). By enriching DE genes which are up-regulated and overlap both comparisons, pathways that exhibit an increase in expression as the MOIs increase were identified (Fig. 6B). The same method was applied to down-regulated genes. However, no continuously down-regulated pathways were identified. The top four up-regulated pathways highlight similar host immune regulated functions that include *TNF signalling*, *NF-κB signalling*, *NOD-like receptor signalling* and *Cytokine-cytokine receptor interaction*; with the proinflammatory cytokine TNF exhibiting almost double the combined score of the next highest. We had anticipated seeing a strong immune-based response, as these pathways are associated with primary defence mechanisms; so, they should increase as the bacterial threat increases.

To further examine influential genes underlying these pathways, ‘trended-genes’ were extracted. The criteria consisted of an expression profile that at least doubled (fold-change > 2) for each comparison, in addition to showing a continued increase from an MOI of 0.1 to 10. In total, 46 genes were identified that trended-upwards (Fig. 6C), no genes trended downwards. These trended-genes further highlight that the underlying host-mechanisms to increased infection at initial stages are predominately immune system associated 24/46 (52%), encompassing cytokine signalling, chemokines and interleukins.

The number of DE genes at 24 h show an even distribution of fold-changes compared to 1 h, with 49% up-regulated comparing MOIs 0.1 and 1, and 50% comparing 1 and 10 (Fig. 6D). Enriched pathways that are continuously up-regulated include *TNF signalling* and *NF-κB signalling*, which are the same top two pathways found at 1 h, and strongly linked to inflammation^31^. We also see two enriched pathways that become down-regulated as the MOI increases: *Carbon metabolism* and the *Citrate cycle (TCA cycle)* (Fig. 6E). This decrease in key metabolism is likely due to cells prioritising defence over growth as the infection escalates.

Examining trended-genes at 24 h uncovers 1 gene exhibiting decreased expression (TXNIP), and 14 genes with increased expression (Fig. 6F). TXNIP (Thioredoxin Interacting Protein) is a thiol-oxidoreductase involved in redox regulation which protects cells against oxidative stress^32^. Chlamydial-specific studies have identified an increase in reactive oxygen species (ROS) at early time points, but expression is rapidly reduced shortly afterwards^33^. A further study has suggested that the redox state within a cell could be a regulator in *Chlamydia*-induced apoptosis^34^. However, it is difficult to know if this decreased regulation is directly linked to chlamydial infection and what advantages an oxidative cellular environment would provide at this developmental stage. Genes with increased expression fall into three main categories: cytokines and inflammation (6 genes), viral-based immune response (5 genes), and ubiquitin-related immune responses (3 genes). As anticipated and seen at 1 h, expression of key immune related genes increases with an increased burden. Only 4 genes overlapped both time points that also increased expression across MOIs (CXCL1, CXCL2, CXCL8 and IL6), indicating their importance as immune mediators against infection.

### Comparative analysis of chlamydial expression between MOIs

**Figure 7:**
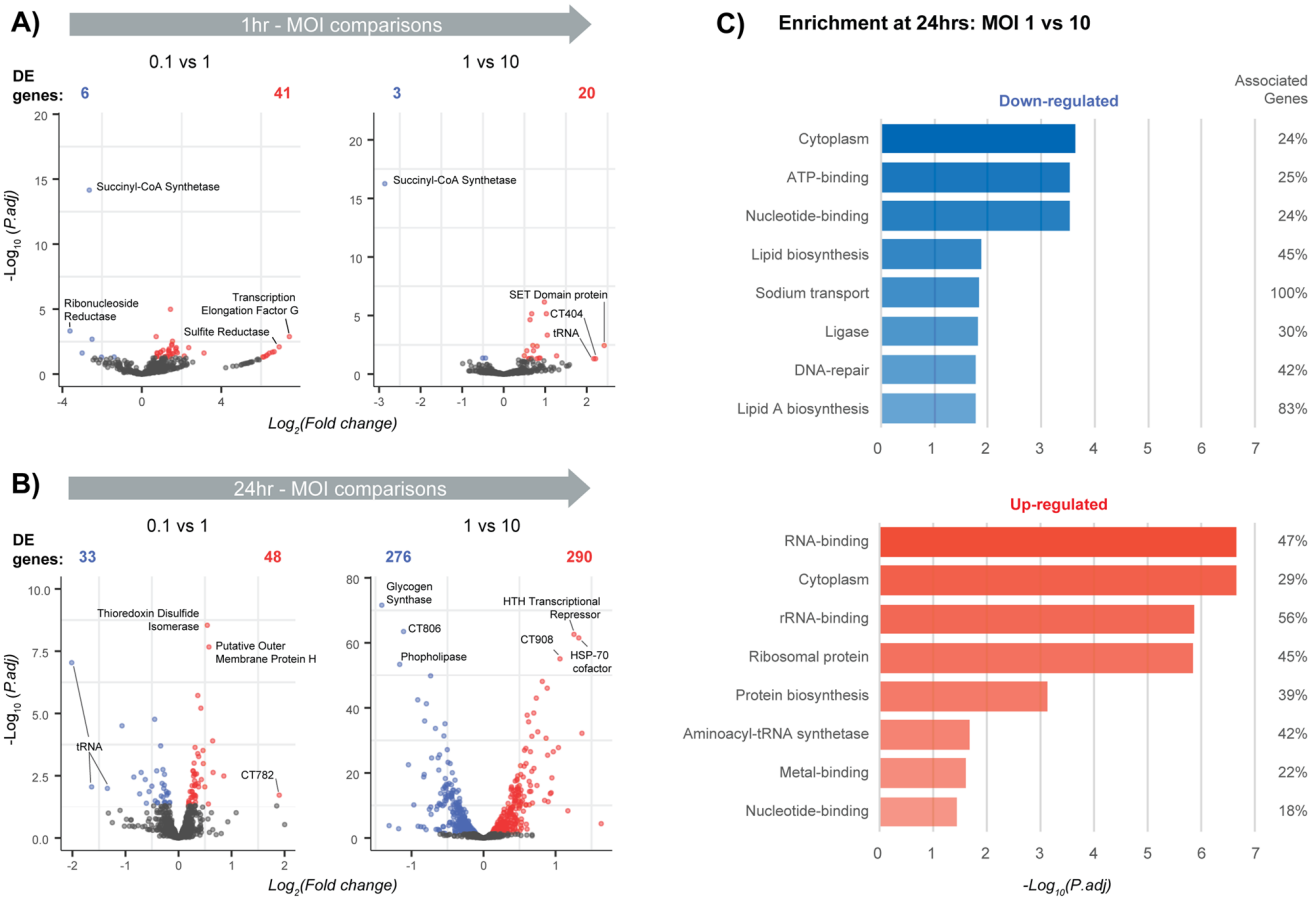
Comparison of differentially expressed chlamydial genes across MOIs. (**A**) Volcano plots after differential comparisons between MOIs, blue dots are down-regulated and red dots are up-regulated (FDR < 0.05). At 1 h, a low number of differentially expressed (DE) genes is observed. (**B**) At 24 h, a slight increase in DE genes is seen comparing MOIs 0.1 and 1, whereas DE genes increase considerably comparing MOIs 1 and 10. (**C**) At 24 h, the increase in DE genes comparing MOIs 1 and 10 allowed enrichment of up and down-regulated genes.

DE genes were also identified to explore chlamydial-based changes attributed to different MOIs. The number of DE genes at 1 h reflected the underlying minimal expression profiles already identified (Fig. 2), with 47 DE genes comparing MOIs 0.1 and 1, and 23 genes comparing 1 and 10 (Fig. 7A). At 24 h, the increase in underlying expression resulted in an increase in DE genes, with 81 comparing MOIs 0.1 and 1, while over half (56%) of the chlamydial genome (566/1008 genes) showed a significant change in regulation comparing MOIs 1 and 10 (Fig. 7B).

No chlamydial genes continuously increased across MOIs at either time point. Only two genes showed a continued decreased in expression: SCLA1|TEF25 (Succinyl-CoA Synthetase) at 1 h, and CT726 (tRNA) at 24 h. The decrease of transfer RNAs (tRNA) at 24 h is slightly surprising, considering they are an important component of translation, and would likely be in abundance during this growth phase of the developmental cycle. Also surprising is a decrease in Succinyl-CoA synthetase, which is involved with the citric acid cycle and cellular metabolism^35^. We can theorise from these genes, as more EBs are introduced, the likelihood of multiple infections within a cell is greatly increased and perhaps some inclusions are benefitting from effector proteins already circulating within the cell from existing inclusions.

Due to low numbers of DE genes at 3 of the 4 comparisons, enrichment was only possible comparing MOIs 1 and 10 at 24 h (Fig. 7C). Down-regulated functions comprise genes that show decreased expression at a higher MOI. Results also show unexpected functions such as *ATP-binding* and *Lipid biosynthesis*, which would generally be associated with chlamydial growth. This may highlight the possibility that inclusions may benefit from effector proteins already in existence, likely reducing the need to express these genes and associated processes. Up-regulated genes cover a wider range functions, with half associated with different binding mechanisms facilitating transcription and growth (*RNA-binding*, *rRNA-binding*, *Metal-binding* and *Nucleotide-binding*); which is expected at this stage of the developmental cycle, especially with a ten-fold increase in EBs.

## Discussion

There is a finite balance when infecting monolayers to accurately measure both host-cell and chlamydial transcriptional responses. This experiment used the universally standard MOI of 1, in addition to a ten-fold increase (MOI 10) and decrease (0.1), to directly observe what changes occur. One reason to increase the MOI is to examine early time points of infection when chlamydial transcripts are in low quantities as seen in (Fig. 2A). In this experiment, when increasing the MOI to 10 at both time points, an increased capture rate of chlamydial transcripts was observed, confirming the suitability for early times (Fig. 2A,B). However a challenge when working with higher MOIs is that some cells may have formed multiple inclusions which may skew host-cell responses beyond what may be seen in a real-world infection setting^36^. When looking at an MOI of 10 at 24 h, we see over 60% of total captured transcripts from *Chlamydia*. Although this may not be representative of an in vivo infection, it is highly useful when focusing on chlamydial-based mechanisms. However, this does raise a question regarding a theoretical maximum proportion of chlamydial reads that can exist within a host cell during the developmental cycle, particularly at the later stages of infection. This was highlighted from (Fig. 2E) at 24 h, where we see a single replicate showing a staggering 74% of all transcripts associated with chlamydial expression. What was difficult to determine was if the increase in EBs had a corresponding influence in reducing the length of the developmental cycle due to possible synergistic interactions and shared resources. A future dual RNA-seq study following the time course of (Miyairi et al., 2006), but replacing biovars for MOIs would be intriguing.

We know from existing studies that different MOIs need to be used when examining different stages of the developmental cycle. For example, early time points generally require a higher MOI as limited transcription from *Chlamydia* occurs, resulting in low capture rates that can be difficult to interpret^19,20^. During mid-stages, an MOI of 1 is often used to capture events based around growth and replication^37^. Towards the latter stages, almost all chlamydial genes are transcribed, making biological interpretations challenging^15^. Most studies examining a range of developmental stages use an MOI of 1 and this has generally been considered suitable. However, MOIs are generated from serial dilutions, so lower MOIs such as 1, may not actually have 1 EB per cell. Results from this experiment show a substantial increase in capture rates and transcription from an MOI of 1 to 10, suggesting that a slightly higher MOI may be optimal. There are however implications that need to be taken into consideration when using MOIs higher than 1. These include that EBs preferentially infect cells together rather than spread out evenly, which can result in all variations of the intended MOI. For example, a starting MOI of 5 will likely see an MOI range between 0 and 5 across a population of cells. As a result, the overall captured signal may be difficult to interpret, particularly with large MOIs. Furthermore, the length of the developmental cycle is generally shortened when many EBs are internalised due to an increased burden on the host cell.

By combining rRNA and polyA depletion methods, we clearly observe an increased capture rate of chlamydial transcripts. These additional transcripts do not appear to be from a small subset of genes dominating capture, but from a wide range expressed genes (Fig. 3). However, host-based expression is affected, with expression of non-protein coding genes increasing (Fig. 4); suggesting it may only be beneficial for future chlamydial-specific sequencing approaching to use both depletion methods.

Unfortunately, this experimental design did not include any mock infected replicates from either time point, limiting some analyses. Future experiments would benefit from their inclusion, helping to separate general cell proliferation events from infection-relevant results.

A further limitation was not having the ability to determine if the timeframes of the developmental cycle are affected relative to increasing the MOI. The possibility of this occurring is quite likely, particularly as more EBs are internalised, resulting in more inclusions putting an increased burden on the host cell. Perhaps a future experiment could include a fluorescent tag that could be quantified as cells become lysed, thereby providing a measurement relative to the MOI and length of the developmental cycle. Alternatively, chemical-assisted methods can arrest at different cell cycle stages, ensuring all cells are synchronised at a specified cell cycle phase and removing this as a potential confounding factor.

Differentially expressed and trended genes (Fig. 6) identified transcriptional responses the host cell uses during an infection, which appears to be from a similar subset of key genes at both times. Genes are associated with immune related pathways, specifically inflammation; with increased expression as the MOI increases. As the concentration of EBs increases provoking this increased immune response, host cells will likely become overwhelmed if the numbers of EBs become too high. We hypothesise this could be an advantage for *Chlamydia* if a large proportion of host cell expression is focused towards immune responses, and if they already have a way of countering these, then other host processes may be easier to interfere with and possibly hijack. We anticipate this would most likely occur at higher MOIs where we have observed the most difference, particularly at 24 h or latter stages of the developmental cycle.

## Conclusion

This work highlights how future bacterial-specific RNA-seq studies can increase sequence capture rates by combining rRNA and polyA depletion methods. This is particularly relevant for chlamydial-based expression studies when examining early time points, as low expression is generally observed. Three different MOIs highlighted that significantly more Chlamydial transcripts were captured at both time points when using an MOI of 10. Although useful for capturing Chlamydial-specific biology, the increased burden on host cells may not be representative of in vivo infections. Overall, these outcomes can help influence future NGS-based experimental designs to achieve more specific infection-related biological outcomes, particularly from *Chlamydia*-infected cells.

## Methods

### Cell culture and infection

Human epithelial type 2 (HEp-2) cells (American Type Culture Collection, ATCC No. CCL-23) were grown as monolayers in 6 × 100 mm tissue culture dishes until cells were 90% confluent. To harvest EBs for the subsequent infections, additional monolayers were grown and infected with *C. trachomatis* serovar E in sucrose phosphate glutamate (SPG) as previously outlined^38^. The resulting EBs and cell lysates were then harvested and used to infect new HEp2 monolayers.

Infections for each dataset used the previously prepared HEp2 monolayers, infecting with *C. trachomatis* serovar E in 3.5 mL SPG buffer as previously outlined^38^; infections were synchronised using centrifugation. EBs were introduced into monolayers from three MOIs (0.1, 1 and 10) using 1:10 dilutions beginning from an MOI of 10. EBs were quantified as previously described^15^. To remove non-viable or dead EBs, each sample was incubated at 25 °C for 2 h, and washed twice in SPG. Cell monolayers were incubated at 37 °C with 5% CO_2_, including the addition of 10 mL fresh medium (DMEM + 10% FBS, 25 μg/ml gentamycin, 1.25 μg/ml Fungizone). After each infection time point, the infected and uninfected dishes were harvested by scraping and resuspending in 150 μL sterile PBS. Any resuspended samples were stored at − 80 °C.

### Library preparation and sequencing

Ribo-Zero rRNA Removal kits (Human/Mouse/Rat and Gram-negative) were used to deplete samples of both human and gram-negative bacterial rRNA. Equivalent volumes from each kit were combined, thereby allowing the removal of bacterial and human rRNA simultaneously within each sample. Each sample was equally separated, with one half subjected to polyA depletion by the Poly(A) Purist Mag purification kit (Ambion), whereby removing host-based polyA transcripts to allow the enrichment of bacterial transcripts. Magnetic beads were used to bind to polyA mRNAs and were extracted from the solution with a magnet. Samples with combined depletion methods were further purified using Zymo-Spin IC columns (Zymo Research) before being re-combined for library construction.

The mRNA libraries were prepared from depleted samples as previously stated at 1 and 24 h post infection (times are calculated up to the point of lysis), using the TruSeq RNA Sample Prep kit (Illumina, San Diego, CA) per the manufacturer's protocol with IGS-specific optimisations. Adapters and indexes (6 bp) were ligated to the double-stranded cDNA, which was subsequently purified with AMPure XT beads (Beckman Coulter Genomics, Danvers, MA) between enzymatic reactions and size selection steps (∼ 250 to 300 bp). The resulting libraries were sequenced on an Illumina HiSeq2000 using the 100 bp paired-end protocol at the Genome Resource Centre, Institute for Genome Sciences, University of Maryland School of Medicine.

### Bioinformatic analysis

Sequencing reads were trimmed and quality checked using Trim Galore (0.45) (https://www.bioinformatics.babraham.ac.uk/projects/trim_galore/) and FastQC (0.11.5)^39^. Host reads were aligned to the human genome (GRCh 38.87) using STAR (2.5.2b)^40^, while chlamydial reads were aligned to the *Chlamydia trachomatis* (serovar E, Charm001) genome using Bowtie2 (2.3.2)^41^ with additional parameters of ‘1 mismatch’ and ‘–very-sensitive-local’. Samtools (1.6)^42^ was used to remove duplicate reads in addition to only keeping mapped reads in both the host and chlamydial BAM files. To remove reads that mapped to both genomes, we first extracted the mapped reads back into paired-end fastq files using BEDtools (2.26.0)^43^. Reads were then aligned using the initial mapping software to the reciprocal genomes. Any reads that mapped to both genomes were removed from the originating BAM files using the ‘FilterSamReads’ command from Picard tools (2.10.4)^44^. Additional quality control metrics were examined using BEDtools (2.26.0)^43^, Bamtools (2.5.1)^45^, MultiQC (1.2)^46^ and various in-house scripts.

Features (genes) were counted using featureCounts (1.5.0-p1)^47^ with additional parameters of ‘-Q 10 -p -C’. Genefilter (1.64.0)^48^ was used to filter out genes with low counts, where host genes were retained if expression > 50 in at least three samples. To accommodate the vast differences in expression between host and chlamydial reads, a separate filter was used retaining chlamydial genes with expression > 10 in at least three samples. Chlamydial and host reads were further separated by time point due to the large amount of variability in expression between an MOI of 0.1 at 1 h to an MOI of 10 at 24 h. Once separated, library normalisation was performed using the trimmed mean of M-values (TMM) method^49^.

To identify outliers, four PCA bi-plots were generated from library normalised counts using PCATools (0.99.13)^50^, where eigenvalues from PC1 and PC2 for each replicate were calculated and used to highlight outlier samples if an eigenvalue was >|3| standard deviations from the mean within that group. If an outlier was removed, eigenvalues were recalculated and the process repeated until no further outliers were detected. To determine the underlying variation at each principal component, the “plotloadings” function within PCATools^50^ was used.

Differential expression was performed with edgeR (3.24.3)^51^, adding the difference between the depletion methods as a blocking factor, whereby allowing MOI and time point comparisons to utilise all six replicates to increase significance. Host DE genes were uploaded and enriched for KEGG pathways using the Enrichr database^52^. Relevant host pathways were determined using the combined score with a cutoff of > 50. Combined scores were calculated by adding together the combined scores comparing MOIs 0.1 vs 1, and 1 vs 10. Enrichment of chlamydial DE genes was performed using STRING (11.0)^53^.

Pairwise comparisons were calculated using a two-sided Students t-test and base functions in R with significance set at < 0.05. Both groups were examined to ensure the underlying statistical assumptions were met, which included checking if the data is normally distributed (Shapiro–Wilk test) and comparing the mean and variances (F-test).

## Supplementary Information

Supplementary Information.

Supplementary File 1.

Supplementary File 2.

Supplementary Table 1.

Supplementary Table 2.

Supplementary Table 3.

Supplementary Table 4.

## Data Availability

Transcriptome sequencing data and count matrices are available in the NCBI Gene Expression Omnibus (NCBI GEO) and are accessible through the BioProject PRJNA630978 and within the Short Read Archive (SRA) accession SRP260442.

## Code availability

The underlying source code used for all analyses is available on GitHub https://github.com/reganhayward/Manuscripts-code.

## References

[1] Westermann AJ, Gorski SA, Vogel J (2012). Dual RNA-seq of pathogen and host. Nat. Rev. Microbiol..

[2] Mika-Gospodorz B (2020). Dual RNA-seq of *Orientia tsutsugamushi* informs on host–pathogen interactions for this neglected intracellular human pathogen. Nat. Commun..

[3] Pisu D, Huang L, Grenier JK, Russell DG (2020). Dual RNA-Seq of Mtb-infected macrophages in vivo reveals ontologically distinct host–pathogen interactions. Cell Rep..

[4] Nuss AM (2017). Tissue dual RNA-seq allows fast discovery of infection-specific functions and riboregulators shaping host–pathogen transcriptomes. Proc. Natl. Acad. Sci..

[5] Westermann AJ (2016). Dual RNA-seq unveils noncoding RNA functions in host–pathogen interactions. Nature.

[6] Rienksma RA (2015). Comprehensive insights into transcriptional adaptation of intracellular mycobacteria by microbe-enriched dual RNA sequencing. BMC Genomics.

[7] Baddal B (2015). Dual RNA-seq of nontypeable *Haemophilus influenzae* and host cell transcriptomes reveals novel insights into host–pathogen cross talk. MBio.

[8] Schachter J, Caldwell HD (1980). Chlamydiae. Annu. Rev. Microbiol..

[9] Reyburn H (2016). WHO guidelines for the treatment of *Chlamydia trachomatis*. WHO.

[10] Burton MJ, Mabey DCW (2009). The global burden of trachoma: A review. PLoS Negl. Trop. Dis..

[11] Brunham RC, Binns B, McDowell J, Paraskevas M (1986). *Chlamydia trachomatis* infection in women with ectopic pregnancy. Obstet. Gynecol..

[12] Menon S (2015). Human and pathogen factors associated with *Chlamydia trachomatis*-related infertility in women. Clin. Microbiol. Rev..

[13] Ali H (2015). A new approach to estimating trends in chlamydia incidence. Sex. Transm. Infect..

[14] Hafner LM, Wilson DP, Timms P (2014). Development status and future prospects for a vaccine against *Chlamydia trachomatis* infection. Vaccine.

[15] Humphrys MS (2013). Simultaneous transcriptional profiling of bacteria and their host cells. PLoS ONE.

[16] Belland RJ (2003). Genomic transcriptional profiling of the developmental cycle of *Chlamydia trachomatis*. Proc. Natl. Acad. Sci. USA..

[17] Belland RJ (2003). Transcriptome analysis of chlamydial growth during IFN-gamma-mediated persistence and reactivation. Proc. Natl. Acad. Sci. USA..

[18] Albrecht M, Sharma CM, Reinhardt R, Vogel J, Rudel T (2010). Deep sequencing-based discovery of the *Chlamydia trachomatis* transcriptome. Nucleic Acids Res..

[19] Grieshaber S (2018). Impact of active metabolism on *Chlamydia trachomatis* elementary body transcript profile and infectivity. J. Bacteriol..

[20] Wang A (2013). Transcription factor complex AP-1 mediates inflammation initiated by *Chlamydia pneumoniae* infection. Cell Microbiol..

[21] Beaulieu LM (2015). Specific inflammatory stimuli lead to distinct platelet responses in mice and humans. PLoS ONE.

[22] O'Connell CM (2011). Toll-like receptor 2 activation by *Chlamydia trachomatis* is plasmid dependent, and plasmid-responsive chromosomal loci are coordinately regulated in response to glucose limitation by *C. trachomatis* but not by *C. muridarum*. Infect. Immunity.

[23] Johnson RM (2018). B cell presentation of chlamydia antigen selects out protective CD4γ13 T cells: Implications for genital tract tissue-resident memory lymphocyte clusters. Infect. Immun..

[24] Yeung ATY (2017). Exploiting induced pluripotent stem cell-derived macrophages to unravel host factors influencing *Chlamydia trachomatis* pathogenesis. Nat. Commun..

[25] Lyons JM, Ito JI, Peña AS, Morré SA (2005). Differences in growth characteristics and elementary body associated cytotoxicity between *Chlamydia trachomatis* oculogenital serovars D and H and *Chlamydia muridarum*. J. Clin. Pathol..

[26] Miyairi I, Mahdi OS, Ouellette SP, Belland RJ, Byrne GI (2006). Different growth rates of *Chlamydia trachomatis* biovars reflect pathotype. J. Infect. Dis..

[27] Huang C (2017). A snoRNA modulates mRNA 3′ end processing and regulates the expression of a subset of mRNAs. Nucleic Acids Res..

[28] Shutt TE, Shadel GS (2010). A compendium of human mitochondrial gene expression machinery with links to disease. Environ. Mol. Mutagen..

[29] Jiang Y (2018). Co-activation of super-enhancer-driven CCAT1 by TP63 and SOX2 promotes squamous cancer progression. Nat. Commun..

[30] West JA (2014). The long noncoding RNAs NEAT1 and MALAT1 bind active chromatin sites. Mol. Cell.

[31] Lawrence T (2009). The nuclear factor NF-kappaB pathway in inflammation. Cold Spring Harb. Perspect. Biol..

[32] Chutkow WA, Patwari P, Yoshioka J, Lee RT (2008). Thioredoxin-interacting protein (Txnip) is a critical regulator of hepatic glucose production. J. Biol. Chem..

[33] Boncompain G (2010). Production of reactive oxygen species is turned on and rapidly shut down in epithelial cells infected with *Chlamydia trachomatis*. Infect. Immun..

[34] Schoier J, Ollinger K, Kvarnstrom M, Soderlund G, Kihlstrom E (2001). *Chlamydia trachomatis*-induced apoptosis occurs in uninfected McCoy cells late in the developmental cycle and is regulated by the intracellular redox state. Microb. Pathog..

[35] Phillips D, Aponte AM, French SA, Chess DJ, Balaban RS (2009). Succinyl-CoA synthetase is a phosphate target for the activation of mitochondrial metabolism. Biochemistry.

[36] Suchland RJ, Rockey DD, Weeks SK, Alzhanov DT, Stamm WE (2005). Development of secondary inclusions in cells infected by *Chlamydia trachomatis*. Infect. Immun..

[37] Abdelrahman YM, Rose LA, Belland RJ (2011). Developmental expression of non-coding RNAs in *Chlamydia trachomatis* during normal and persistent growth. Nucleic Acids Res..

[38] Tan C (2009). *Chlamydia trachomatis*-infected patients display variable antibody profiles against the nine-member polymorphic membrane protein family. Infect. Immunity.

[39] Andrews, S. *FastQC: A quality control tool for high throughput sequence data*. Available online at: http://www.bioinformatics.babraham.ac.uk/projects/fastqc/ (2010).

[40] Dobin A (2013). STAR: Ultrafast universal RNA-seq aligner. Bioinformatics.

[41] Langmead B, Salzberg SL (2012). Fast gapped-read alignment with Bowtie 2. Nat. Methods.

[42] Li H (2009). The sequence alignment/map format and SAMtools. Bioinformatics.

[43] Quinlan AR, Hall IM (2010). BEDTools: A flexible suite of utilities for comparing genomic features. Bioinformatics.

[44] Picard Toolkit. Broad Institute, GitHub Repository. http://broadinstitute.github.io/picard/ (2019).

[45] Barnett DW, Garrison EK, Quinlan AR, Strömberg MP, Marth GT (2011). BamTools: A C++ API and toolkit for analyzing and managing BAM files. Bioinformatics.

[46] Ewels P, Magnusson M, Lundin S, Käller M (2016). MultiQC: Summarize analysis results for multiple tools and samples in a single report. Bioinformatics.

[47] Liao Y, Smyth GK, Shi W (2014). featureCounts: An efficient general purpose program for assigning sequence reads to genomic features. Bioinformatics.

[48] Gentleman, R., Carey, V., Huber, W., Hahne, F. *genefilter: methods for filtering genes from high-throughput experiments.* R package version 1.72.1 (2021).

[49] Robinson MD, Oshlack A (2010). A scaling normalization method for differential expression analysis of RNA-seq data. Genome Biol..

[50] Blighe, K. & Lun, A. *PCAtools: Everything Principal Components Analysis*. R package version 2.2.0 https://github.com/kevinblighe/PCAtools (2018).

[51] Robinson MD, McCarthy DJ, Smyth GK (2010). edgeR: A Bioconductor package for differential expression analysis of digital gene expression data. Bioinformatics.

[52] Kuleshov MV (2016). Enrichr: A comprehensive gene set enrichment analysis web server 2016 update. Nucleic Acids Res..

[53] Szklarczyk D (2018). STRING v11: Protein–protein association networks with increased coverage, supporting functional discovery in genome-wide experimental datasets. Nucleic Acids Res..

