# Supplementary material for: Dual RNA-seq analysis of in vitro infection multiplicity and RNA depletion methods in Chlamydia-infected epithelial cells
Source: Sci Rep. 2021 May 17;11:10399. doi: 10.1038/s41598-021-89921-x (PMC8128910; doi:10.1038/s41598-021-89921-x)
Supplement: Supplementary file 1 — Supplementary Information. [file 41598_2021_89921_MOESM1_ESM.doc]

# Dual RNA-Seq analysis of *in vitro* infection multiplicity and RNA depletion methods in *Chlamydia*-infected epithelial cells

Regan J. Hayward, Michael S. Humphrys, Wilhelmina M. Huston and Garry S.A. Myers

**Supplementary File 1:**

Correlations of top 50 expressed host and chlamydial genes, separated by time point and MOI.

**Supplementary File 2:**

Functional characterisation from top 25 highly expressed genes (host and chlamydial) that overlap all three MOIs.

**Supplementary Table 1:**

Mapping depth and coverage separated in to host and chlamydial reads

**Supplementary Table 2:**

Proportions of host and chlamydial assigned reads from each sample

**Supplementary Table 3:**

Proportions of protein coding and non-protein coding host genes from each sample

**Supplementary Table 4:**

Breakdown of the proportions of non-protein coding gene expression
