# Supplementary figures and images for: Dual RNA-seq analysis of in vitro infection multiplicity and RNA depletion methods in Chlamydia-infected epithelial cells
Source: Sci Rep. 2021 May 17;11:10399. doi: 10.1038/s41598-021-89921-x (PMC8128910; doi:10.1038/s41598-021-89921-x)

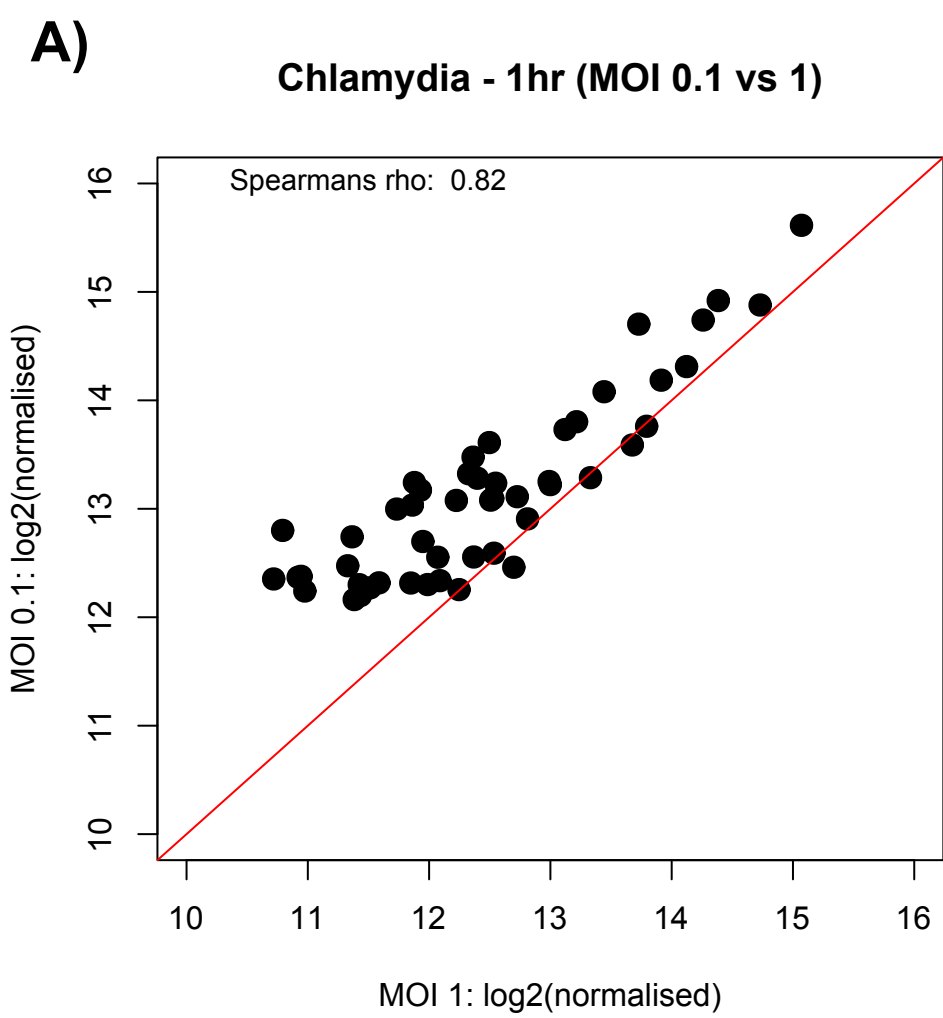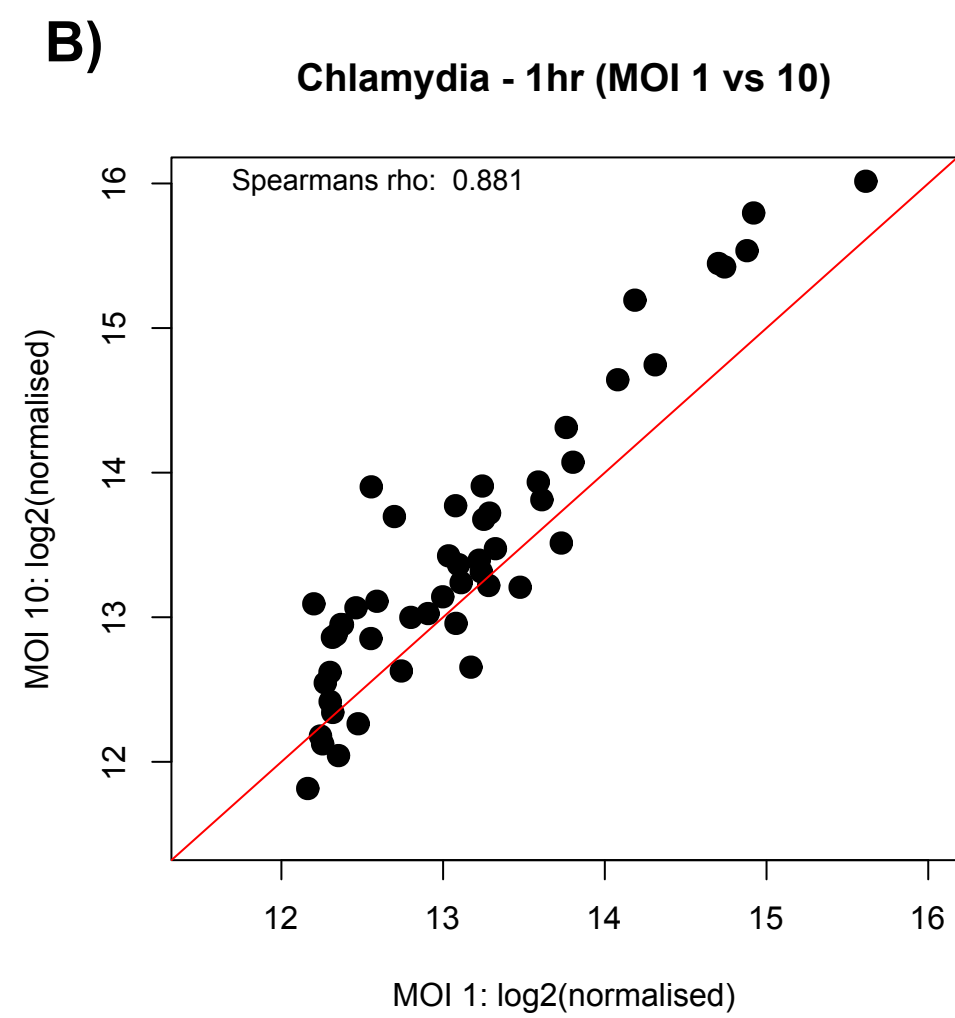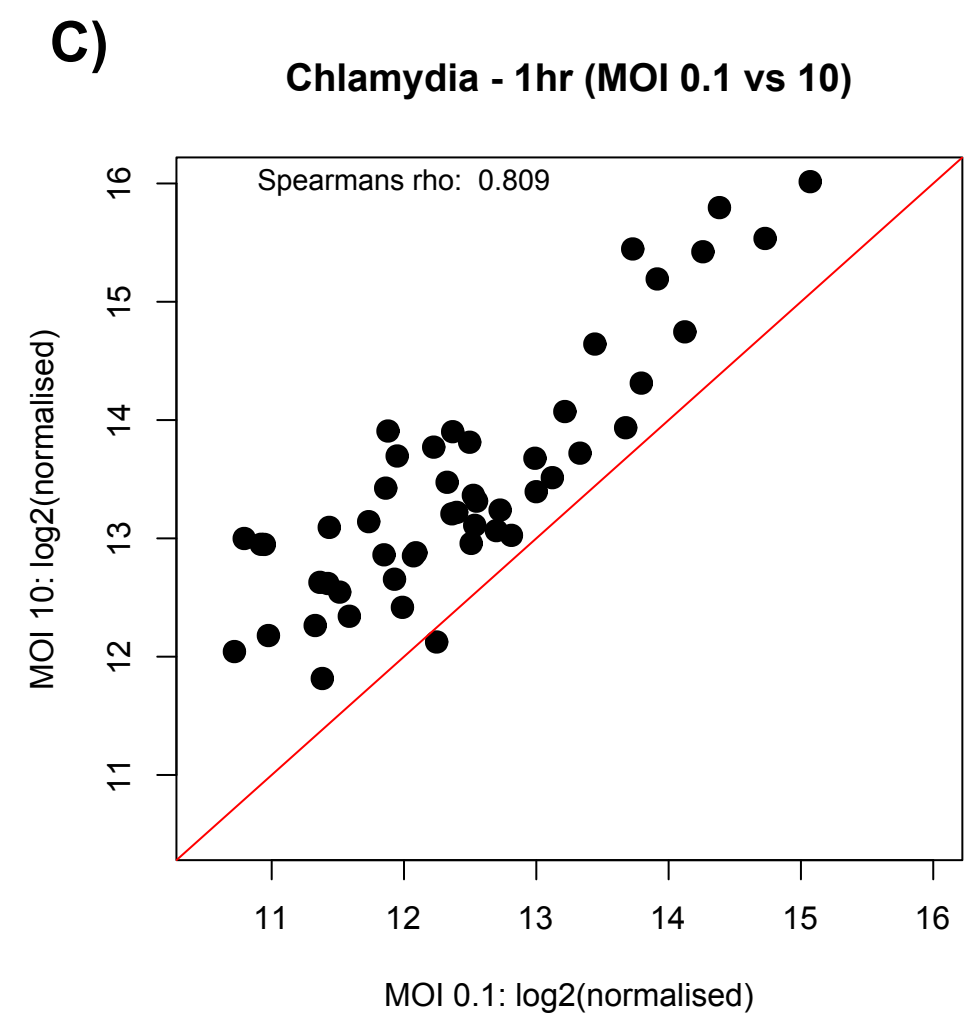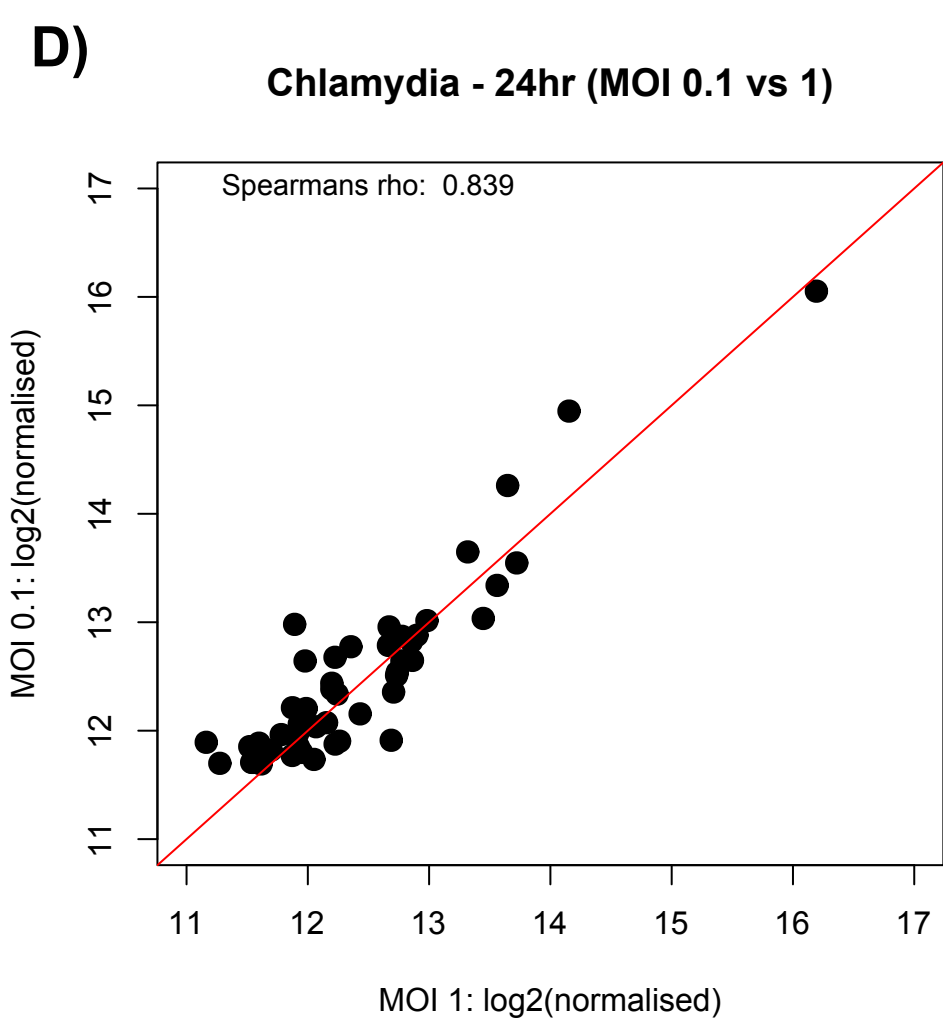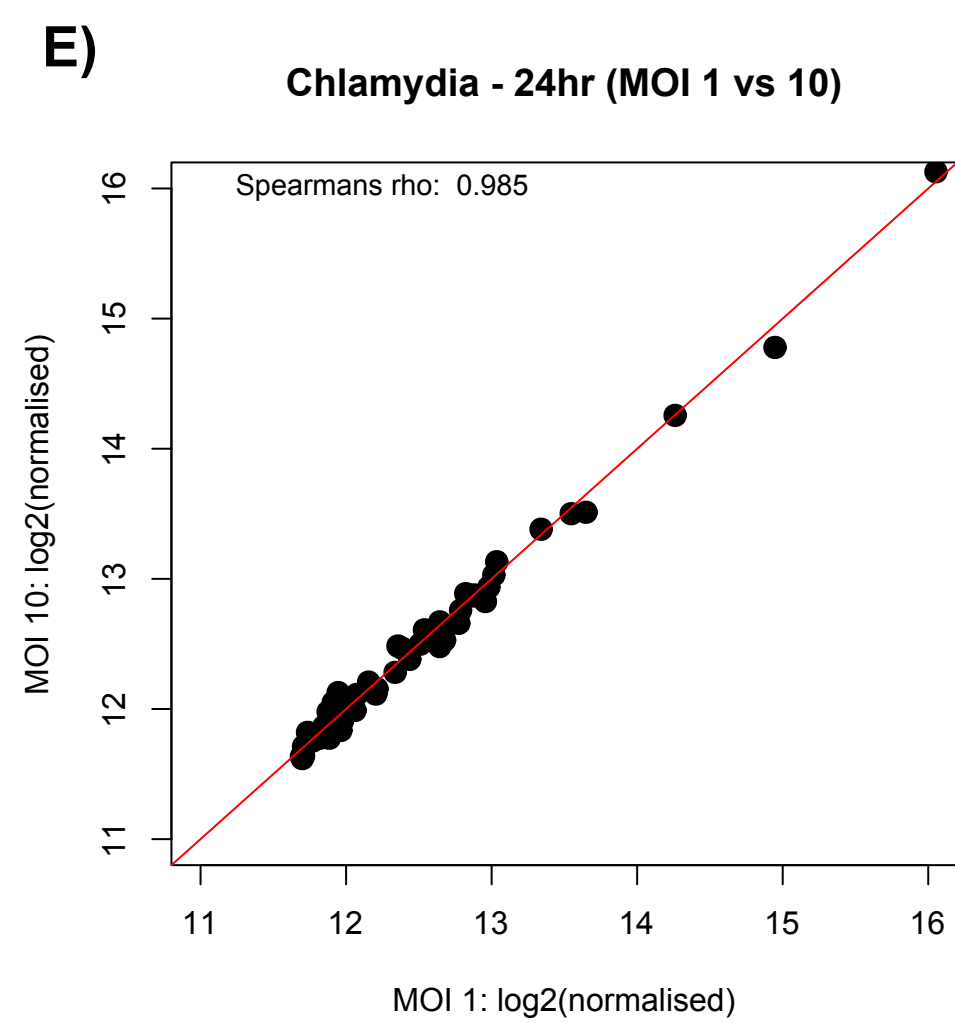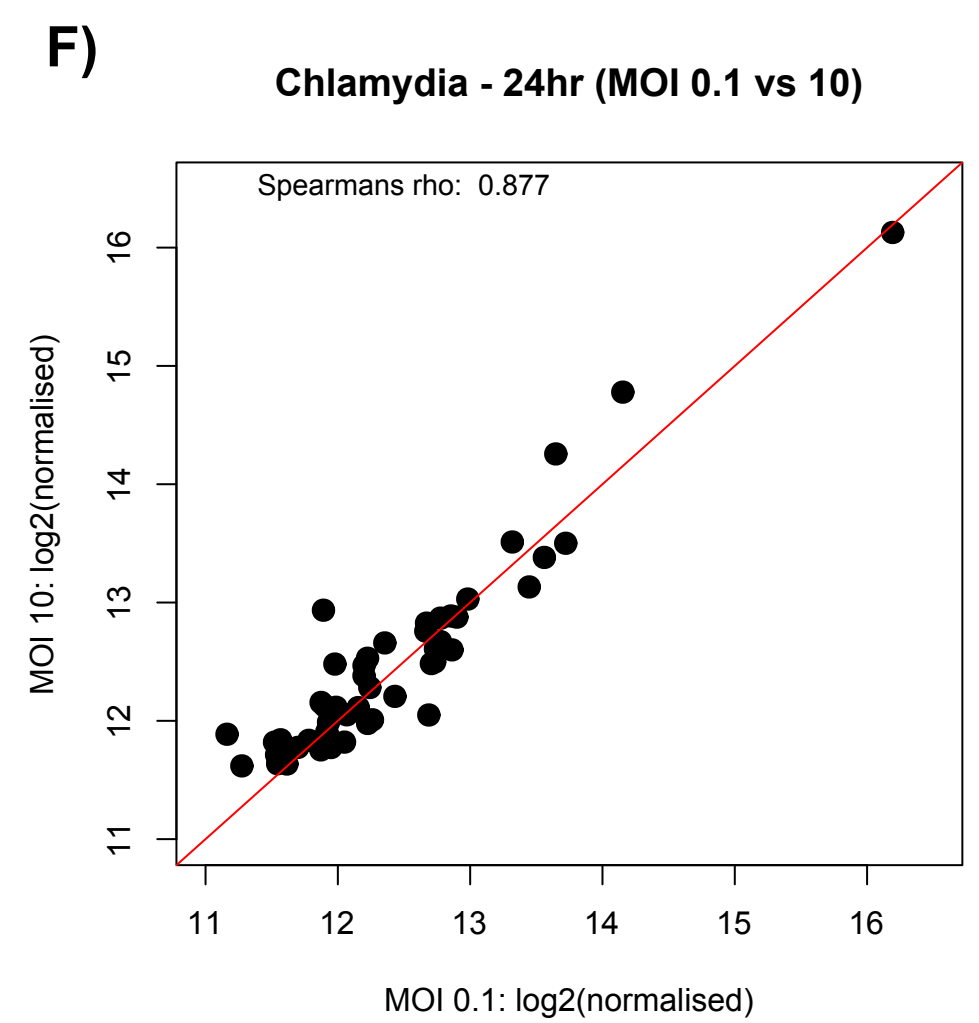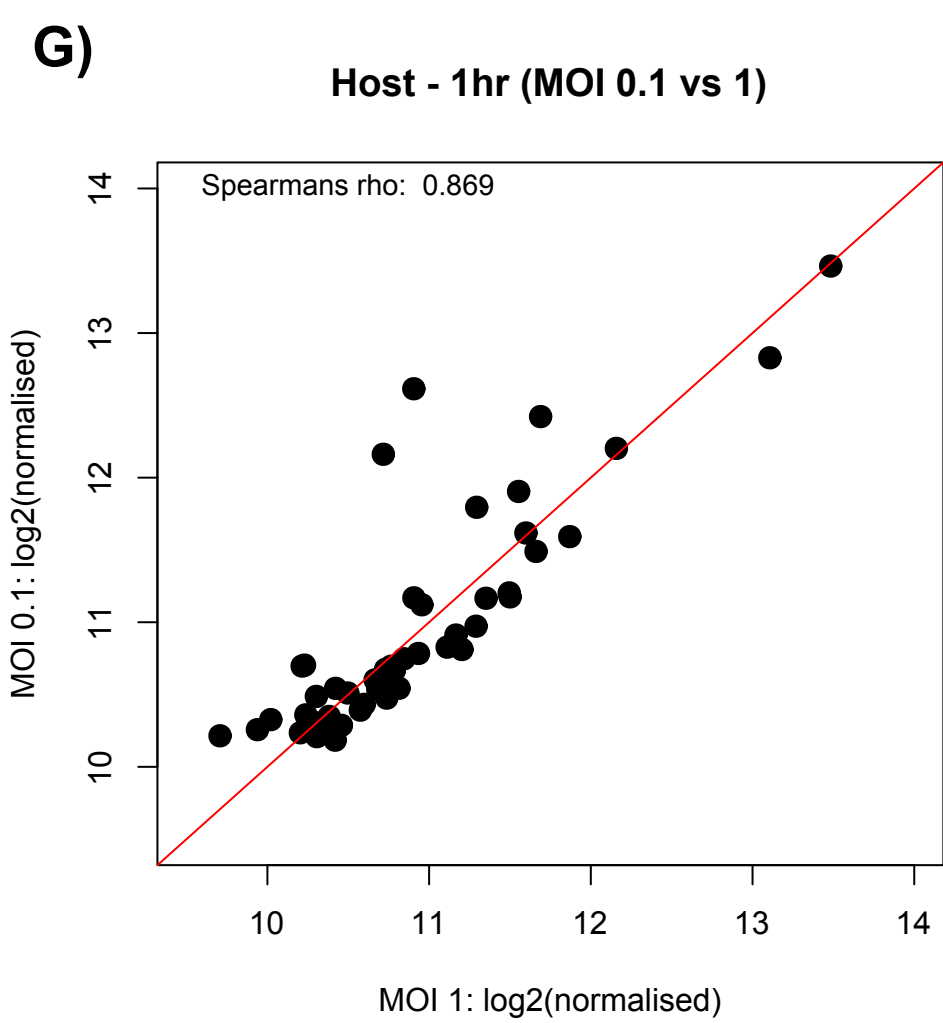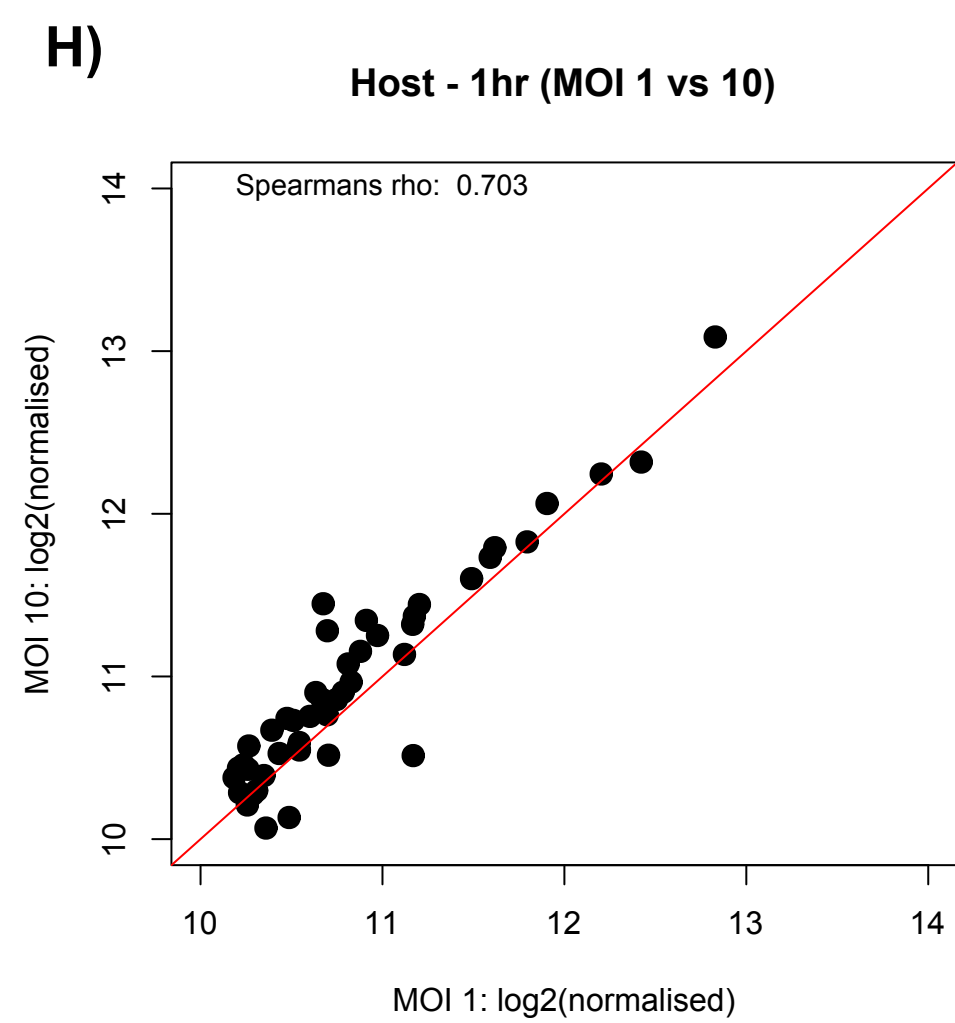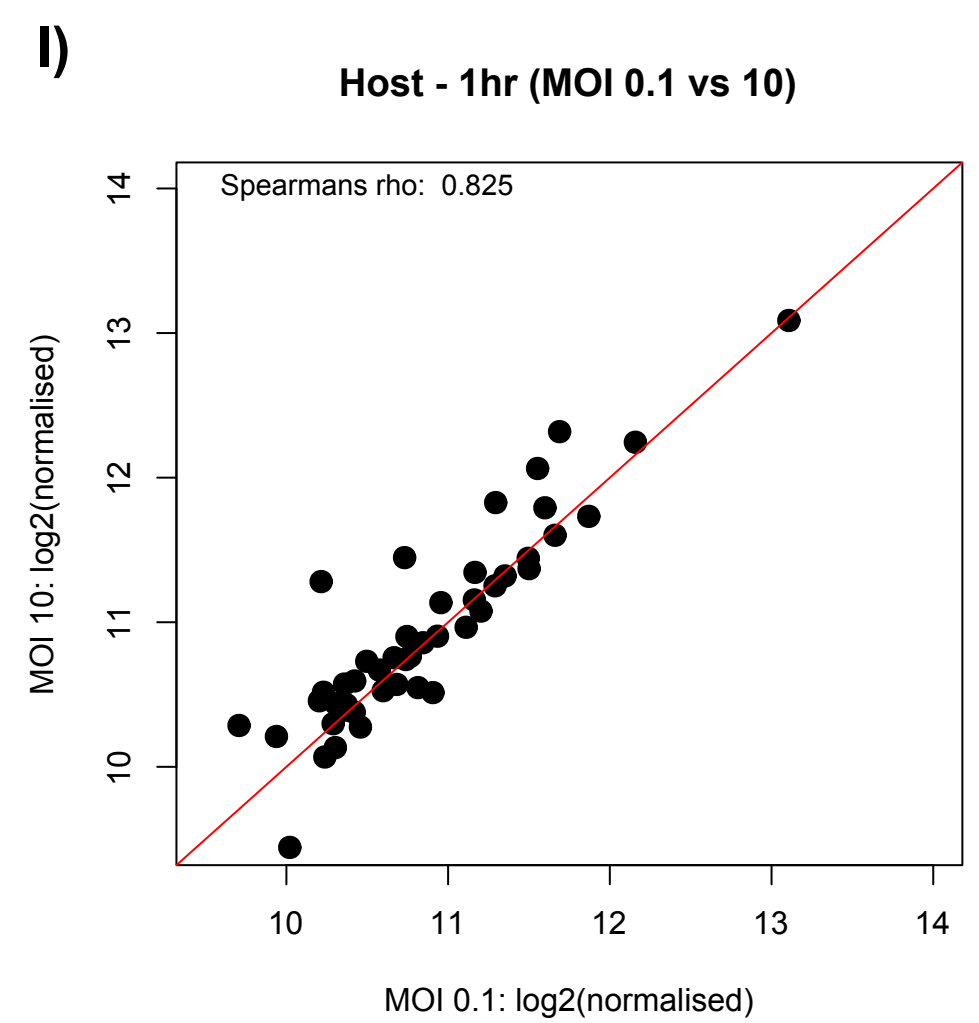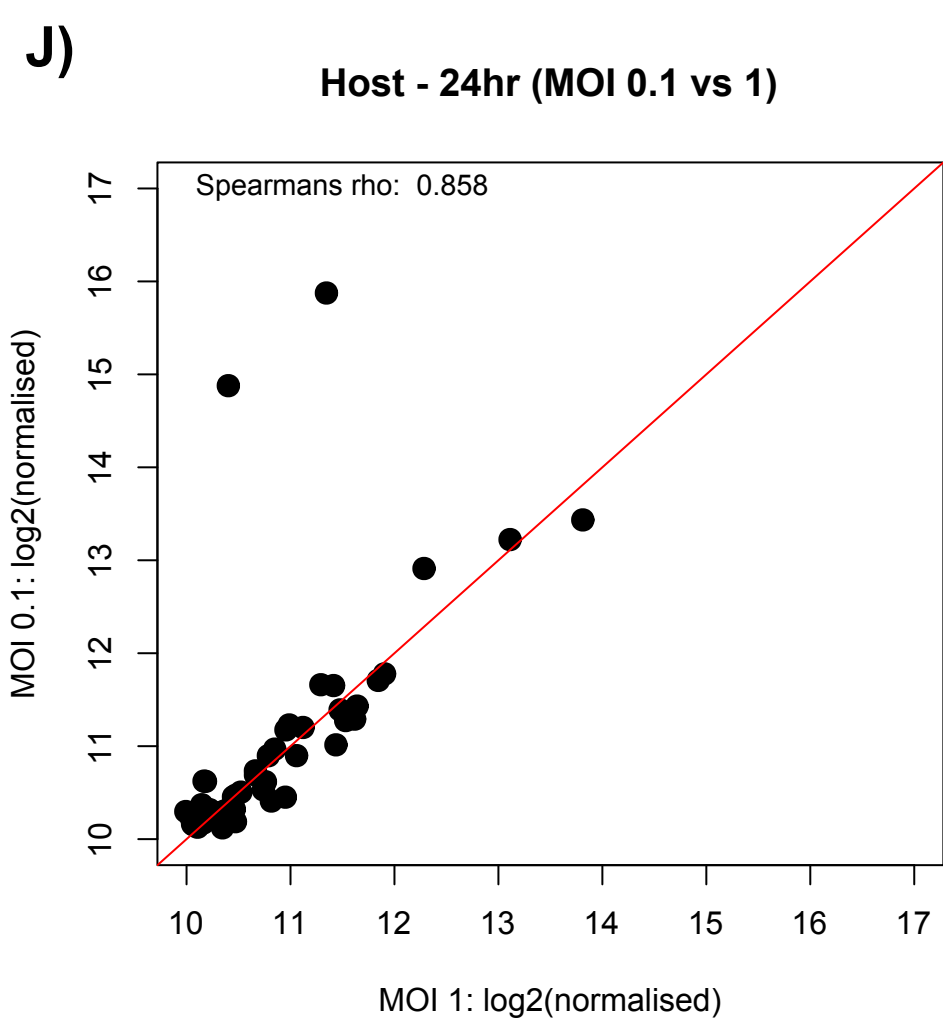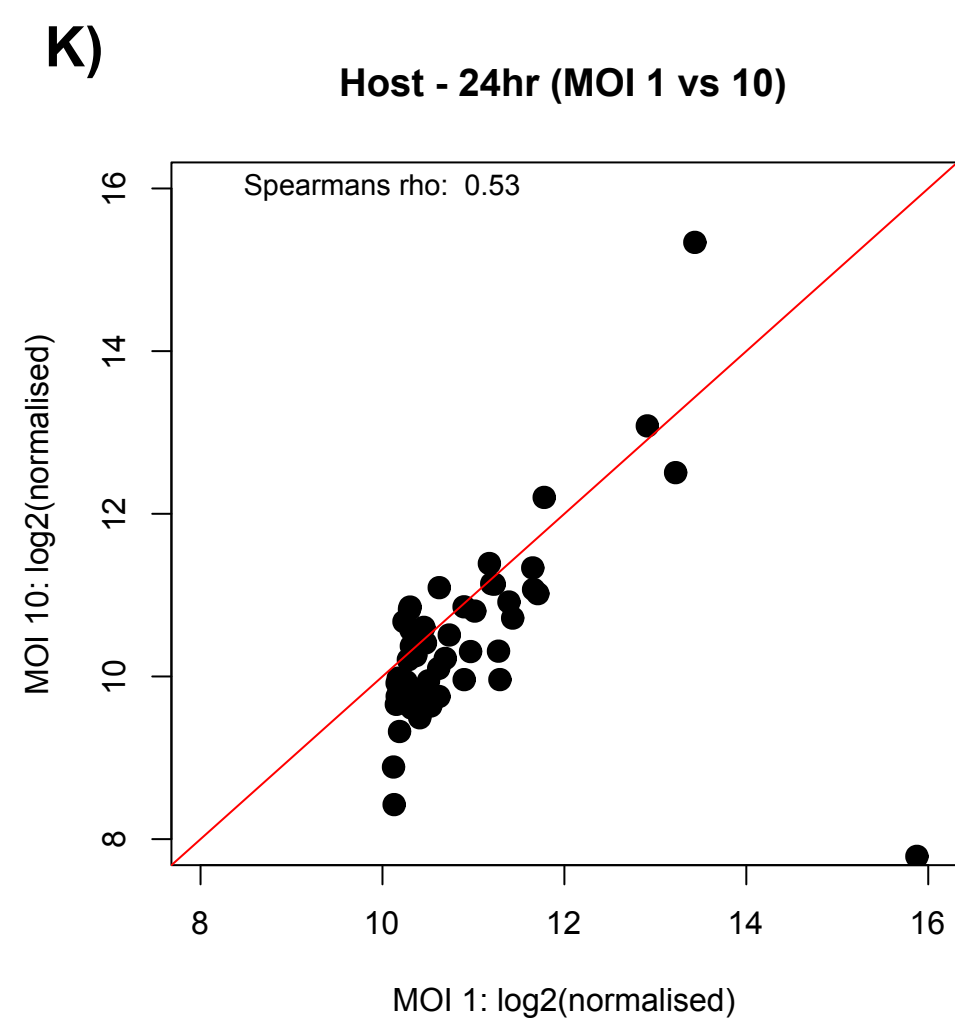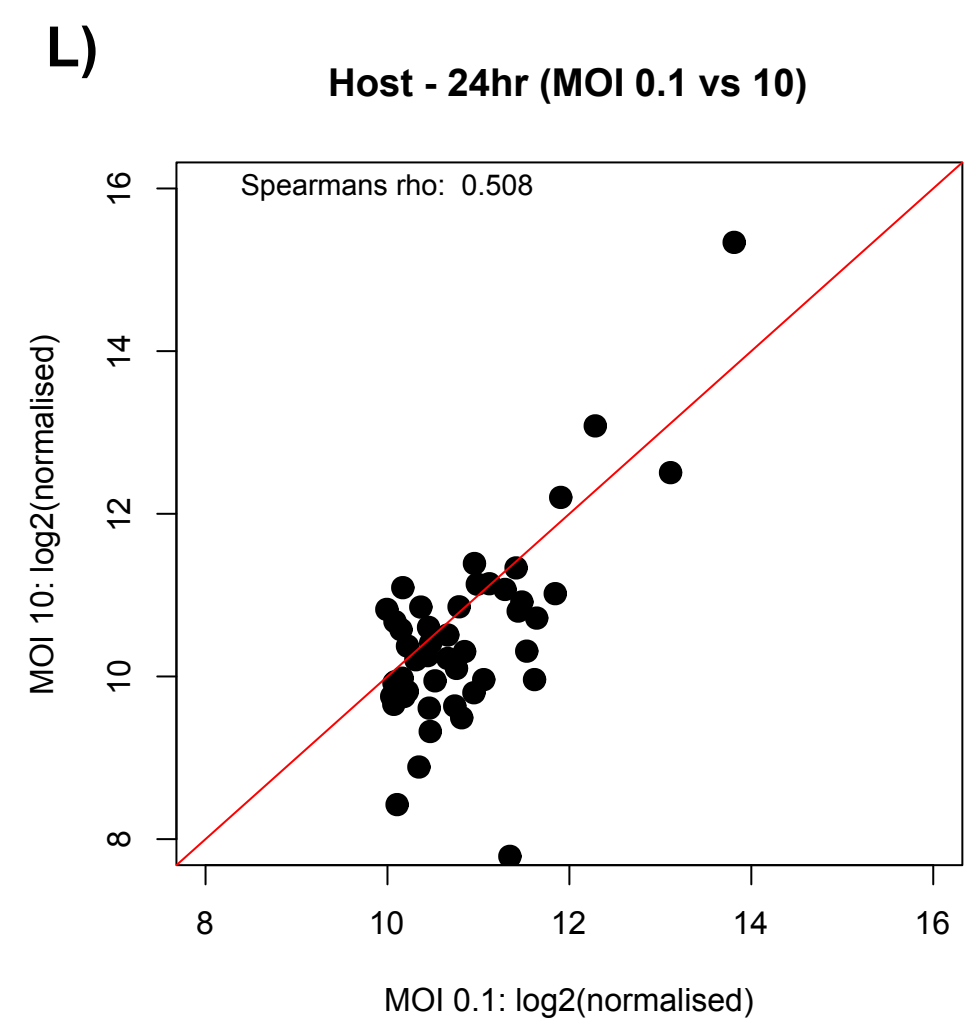

Supplement: Supplementary file 2 — Supplementary File 1. [file 41598_2021_89921_MOESM2_ESM.pdf]
